# Supplementary material for: Shape morphing of hydrogels by harnessing enzyme enabled mechanoresponse
Source: Nat Commun. 2024 Jan 4;15:249. doi: 10.1038/s41467-023-44607-y (PMC10764310; doi:10.1038/s41467-023-44607-y)
Supplement: Supplementary file 1 — Supplementary Information [file 41467_2023_44607_MOESM1_ESM.pdf]

Supplementary Information

Shape Morphing of Hydrogels by Harnessing Enzyme Enabled Mechanoresponse

Kuan Zhang[1,2,3],[5] Yu Zhou[2],[5] Junsheng Zhang[1], Qing Liu[1], Christina Hanenberg[2,3], Ahmed Mourran[2], Xin Wang[1], Xiang Gao[2,3], Yi Cao[1,4], Andreas Herrmann[2,3],[*] Lifei Zheng[1*]

[1] Wenzhou Institute, University of Chinese Academy of Sciences, Wenzhou 325001, China.

[2] DWI – Leibniz-Institute for Interactive Materials, Aachen 52056, Germany.

[3] Institute for Technical and Macromolecular Chemistry, Rheinisch-Westfälische Technische Hochschule (RWTH) Aachen University, Aachen 52074, Germany.

[4] Collaborative Innovation Center of Advanced Microstructures, National Laboratory of Solid State Microstructure, Department of Physics, Nanjing University 22 Hankou Road, Nanjing 210093, China.

[5] These authors contributed equally: Kuan Zhang, Yu Zhou.

[*]Corresponding authors

herrmann@dwi.rwth-aachen.de (A. Herrmann)

zhenglf@ucas.ac.cn (L. Zheng)

Supplementary Note

Materials

The pJET cloning vector, restriction enzymes, T4 DNA ligase (5 Weiss U/µL), and GeneJET Plasmid Miniprep kit were purchased from Thermo Fisher Scientific. Roti®GelStain was received from Carl Roth (Germany). Digested DNA fragments were purified using QIAquick Spin Miniprep kits from QIAGEN (Valencia, CA). Oligonucleotides for sequencing were ordered from Sigma-Aldrich (St. Louis, MO). All peptides used in this work including thrombin-cleavable peptide crosslinker (TCP linker, M_W = 1205.37 g mol^{-1}, purity 97.33 %), peptide crosslinker (M_W = 1304.52 g mol^{-1}, purity 93.33 %) and GR7 (M_W = 708.77 g mol^{-1}, purity 98.83 %) were synthesized by CASLO ApS and purified by High Performance Liquid Chromatography (HPLC). All biochemicals for cloning and protein expression, such as TB medium, salts, antibiotics as well as inducer compounds, were used as received from Sigma-Aldrich or Duchefa. Factor Xa protease was obtained from New England BioLabs. Human Research Grade Fibrinogen was obtained from Cellsystems. All chemicals were of analytical grade and used without further purification unless otherwise mentioned. For all experiments, ultrapure water (18.2 MΩ) purified by a MilliQ-Millipore system (Millipore, Germany) was used.

Protein Sequences

1.Prethrombin 2-SNAP (Prethrombin 2-GS-SNAP)

MHHHHHHIEGRTSEDHFQPFFNEKTFGAGEADCGLRPLFEKKQVQDQTEKEL

FESYIEGRIVEGQDAEVGLSPWQVMLFRKSPQELLCGASLISDRWVLTAAHCL

LYPPWDKNFTVDDLVRIGKHSRTRYERKVEKISMLDKIYIHPRYNWKENLDR

DIALLKLKRPIELSDYIHPVCLPDKQTAAKLLHAGFKGRVTGWGNRRETWTTS

VAEVQPSVLQVVNLPLVERPVCKASTRIRITDNMFCAGYKPGEGKRGDACEG

DSGGPFVMKSPYNNRWYQMGIVSWGEGCDRDGKYGFYTHVFRLKKWIQKV

IDRLGSGGGGSGGGGSGGGGSMDKDCEMKRTTLDSPLGKLELSGCEQGLHRI

IFLGKGTSAADAVEVPAPAAVLGGPEPLMQATAWLNAYFHQPEAIEEFPVPALH

HPVFQQESFTRQVLWKLLKVVKFGEVISYSHLAALAGNPAATAAVKTALSGNP

VPILIPCHRVVQGDLDVGGYEGGLAVKEWLLAHEGHRLGKPGLG

2.SUMO-Hirudin-SNAP (SUMO-Hirudin-GS-SNAP)

MHHHHHHGSDSEVNQEAKPEVKPEVKPETHINLKVSDGSSEIFFKIKKTTPLR

RLMEAFAKRQGKEMDSLRFLYDGIRIQADQAPEDLDMEDNDIIEAHREQIGGV

VYTDCTESGQNLCLCEGSNVCGQGNKCILGSDGEKNQCVTGEGTPKPQSHN

DGDFEEIPEEYLQGGGGSGGGGSGGGGSMDKDCEMKRTTLDSPLGKLELSGC

EQGLHRIIFLGKGTSAADAVEVPAPAAVLGGPEPLMQATAWLNAYFHQPEAIEE

FPVPALHHPVFQQESFTRQVLWKLLKVVKFGEVISYSHLAALAGNPAATAAVK

TALSGNPVPILIPCHRVVQGDLDVGGYEGGLAVKEWLLAHEGHRLGKPGLG

DNA sequence

1.Prethrombin 2-SNAP (Prethrombin 2-GS-SNAP)

ATGCACCATCATCACCATCACATCGAGGGACGCACGTCTGAGGACCATTTC

CAGCCCTTCTTCAACGAGAAGACCTTTGGCGCCGGGGAGGCCGACTGTGG

CCTGCGACCCCTGTTCGAGAAGAAGCAGGTGCAGGACCAAACGGAGAAG

GAGCTTTTCGAGTCCTACATCGAGGGGCGCATCGTGGAGGGTCAGGACGC

GGAGGTTGGCCTCTCGCCCTGGCAGGTGATGCTCTTTCGTAAGAGTCCCCA

GGAGCTGCTCTGTGGGGCCAGCCTCATCAGTGACCGCTGGGTCCTCACGG

CTGCCCACTGTCTCCTGTACCCGCCTTGGGACAAGAACTTCACCGTGGATG

ACCTGCTGGTGCGCATCGGCAAGCACTCCCGCACCAGGTATGAGCGGAAG

GTTGAAAAGATCTCCATGCTGGACAAGATCTACATCCACCCCAGGTACAAC

TGGAAGGAGAATCTGGACCGGGACATCGCCCTGCTGAAGCTCAAGAGGCC

CATCGAGTTATCCGACTACATCCACCCCGTGTGCCTGCCCGACAAGCAGAC

AGCAGCCAAGCTGCTCCACGCTGGGTTCAAAGGGCGGGTGACGGGCTGG

GGCAACCGGAGGGAGACGTGGACCACCAGCGTGGCCGAGGTGCAGCCCA

GCGTCCTCCAGGTGGTCAACCTGCCTCTCGTGGAGCGGCCCGTGTGCAAG

GCCTCCACCCGGATCCGCATCACCGACAACATGTTCTGTGCCGGTTACAAG

CCTGGTGAAGGCAAACGAGGGGACGCTTGTGAGGGCGACAGCGGGGGAC

CCTTCGTCATGAAGAGCCCCTATAACAACCGCTGGTATCAAATGGGCATCGT

CTCATGGGGTGAAGGCTGTGACAGGGATGGAAAATATGGCTTCTACACACA

CGTCTTCCGCCTGAAGAAGTGGATACAGAAAGTCATTGATCGGTTAGGAAG

TGGTGGAGGCGGTTCAGGTGGAGGTGGCTCTGGTGGTGGCGGATCGATGG

ACAAAGACTGCGAAATGAAGCGCACCACCCTGGATAGCCCTCTGGGTAAG

CTGGAACTGTCTGGTTGCGAACAGGGTCTGCACCGTATCATCTTCCTGGGT

AAAGGAACATCTGCCGCTGACGCCGTGGAAGTGCCTGCCCCAGCCGCAGT

GCTGGGTGGACCAGAACCGCTGATGCAGGCAACAGCATGGCTCAACGCAT

ACTTCCACCAGCCTGAGGCCATCGAGGAGTTCCCTGTGCCAGCGCTGCAC

CACCCAGTGTTCCAGCAGGAGAGCTTCACCCGCCAGGTGCTGTGGAAATT

ACTGAAAGTGGTTAAGTTCGGAGAGGTCATCAGCTACAGCCACCTGGCCG

CACTGGCAGGCAATCCTGCTGCCACCGCAGCCGTGAAGACCGCCCTGAGC

GGTAATCCAGTGCCGATTCTGATTCCATGTCATCGTGTTGTGCAGGGTGATC

TGGATGTGGGCGGTTACGAGGGCGGTCTGGCAGTTAAGGAGTGGTTACTG

GCACATGAAGGTCATAGACTGGGTAAGCCTGGTCTGGGT

2.SUMO-Hirudin-SNAP (SUMO-Hirudin-GS-SNAP)

ATGCACCATCATCACCATCACGGGTCGGACTCAGAAGTCAATCAAGAAGCT

AAGCCAGAGGTCAAGCCAGAAGTCAAGCCTGAGACTCACATCAATTTAAA

GGTGTCCGATGGATCTTCAGAGATCTTCTTCAAGATCAAAAAGACCACTCC

TTTAAGAAGGCTGATGGAAGCGTTCGCTAAAAGACAGGGTAAGGAAATGG

ACTCCTTAAGATTCTTGTACGACGGTATTAGAATCCAAGCTGATCAGGCCCC

TGAAGATTTGGACATGGAGGATAACGATATTATTGAGGCTCACCGCGAACA

GATTGGAGGTGTTGTTTACACCGACTGTACTGAATCCGGACAAAACCTGTG

TTTGTGTGAGGGTTCTAACGTCTGTGGTCAGGGTAACAAATGCATCCTGGG

TTCCGACGGTGAAAAGAACCAATGTGTCACTGGTGAAGGTACCCCAAAGC

CGCAGTCCCACAACGATGGAGATTTCGAAGAAATCCCAGAAGAATATCTGC

AGGGTGGAGGCGGTTCAGGCGGAGGTGGCTCTGGCGGTGGCGGATCGATG

GACAAAGACTGCGAAATGAAGCGCACCACCCTGGATAGCCCTCTGGGCAA

GCTGGAACTGTCTGGGTGCGAACAGGGCCTGCACCGTATCATCTTCCTGGG

CAAAGGAACATCTGCCGCCGACGCCGTGGAAGTGCCTGCCCCAGCCGCCG

TGCTGGGCGGACCAGAGCCACTGATGCAGGCCACCGCCTGGCTCAACGCC

TACTTTCACCAGCCTGAGGCCATCGAGGAGTTCCCTGTGCCAGCACTGCAT

CACCCAGTGTTCCAGCAGGAGAGCTTTACCCGCCAGGTGCTGTGGAAACT

GCTGAAAGTGGTGAAGTTCGGAGAGGTCATCAGCTACAGCCACCTGGCCG

CCCTGGCCGGCAATCCCGCCGCCACCGCCGCCGTGAAAACCGCCCTGAGC

GGAAATCCCGTGCCCATTCTGATCCCCTGCCACCGGGTGGTGCAGGGCGAC
CTGGACGTGGGGGGCTACGAGGGCGGGCTCGCCGTGAAAGAGTGGCTGCT
GGCCCACGAGGGCCACAGACTGGGCAAGCCTGGGCTGGGT

The sequences of compositional segments are indicated by corresponding colors in the protein and DNA sequences.

Synthesis of O6-benzylguanine styrene (BS)

The BS ligand was synthesized according to previous references (see Supplementary Fig. 6).[1-4] The obtained products were verified by NMR spectroscopy.

1. Synthesis of 4-vinylbenzyl alcohol (**1**)

4-Vinylbenzyl chloride (13.02 mmol) and sodium acetate (15.71 mmol) were incubated in DMSO (6 mL) and stirred for 24 h at 45 °C under N_2. Then, the solution was cooled to room temperature and poured into 10 mL of water to terminate the reaction, followed by extraction with ethyl acetate three times. The organic layers were collected and dried using anhydrous $MgSO_4$. After being concentrated under vacuum, the residue was redissolved in ethanol (5 mL), was supplemented with 20% aqueous NaOH solution (5 mL), and heated under reflux for 4 h. After terminating the reaction and extracting again with ethyl acetate, the organic layers were combined, washed three times using brine, and dried with anhydrous $MgSO_4$. After removing the solid, the mixtures were concentrated and then dried under vacuum to obtain the target product as a brown oil with a yield of 95.5%. ^{1}H NMR (400 MHz, $CDCl_3$) spectrum is shown in Supplementary Fig. 7.

2. Synthesis of 1-(2-Amino-7H-purin-6-yl)-1-methyl-pyrrolidinium chloride (**2**)

Guanidine (30 mmol) dissolved in dimethylformamide (DMF, 14 mL) and in

1,2-dichloroethane (50 mL) were added to $POCl_3$ (90 mmol) under stirring, and the resulting mixture was heated at 80 °C for 8 h. The reaction was cooled and then slowly poured into 120 mL water. Then, sodium carbonate (18 g) was added to adjust the pH of the mixture to ~5. After transferring to a separatory funnel, the aqueous layer was separated, and sodium hydroxide (2.38 g) was slowly added to obtain a yellow-brown precipitate, which was washed three times with water and added to acetic acid (12%, 50 mL) and stirred at 70 °C for another 5 h. Then, the reaction was cooled to room temperature and the solid was collected by centrifugation. After washing with water, the obtained solid (3.15 g) was further treated with 30 mL NaOH (10%) for 4 h at room temperature, followed by the neutralization with concentrated HCl to pH 5-7. The intermediate product 6-chloro-guanine (2.26 g) was obtained as a yellow solid.

2.26 g of 6-chloro-guanine was suspended in DMF (90 mL) and heated to 60 °C to dissolve under stirring. Then, the solution was cooled to room temperature, and 1-methylpyrrolidine (3.2 mL) was added and stirred at room temperature for 32 h. Then, acetone (5 mL) was added to precipitate the crude product. After washing with ether (50 mL), the desired product was dried and obtained as a white powder. The product was characterized by ^{1}H NMR (400 MHz, d6-DMSO), as shown in Supplementary Fig. 8.

3. Synthesis of BS **3**

4-Vinylbenzyl alcohol (**1**, 136 mg, 1.02 mmol) was dissolved in anhydrous DMF (10 mL), and potassium *tert*-butoxide (246 mg, 2.19 mmol) was added to the solution.

After stirring for 5 min at room temperature, compound **2** (123 mg, 0.48 mmol) was added to the slurry solution and stiring was continued for 3 h at room temperature. The reaction mixture was filtered, and the filtrate was concentrated under vacuum. The residue was further purified by flash chromatography with a gradient flow eluent of dichloromethane with 1% - 6% methanol yielding the desired product BS (**3**, 68.7 mg, 25.3% yield) as yellow solid. The product was characterized by ^{1}H NMR spectroscopy (400 MHz, d6-DMSO), as shown in Supplementary Fig. 9.

Supplementary Figures

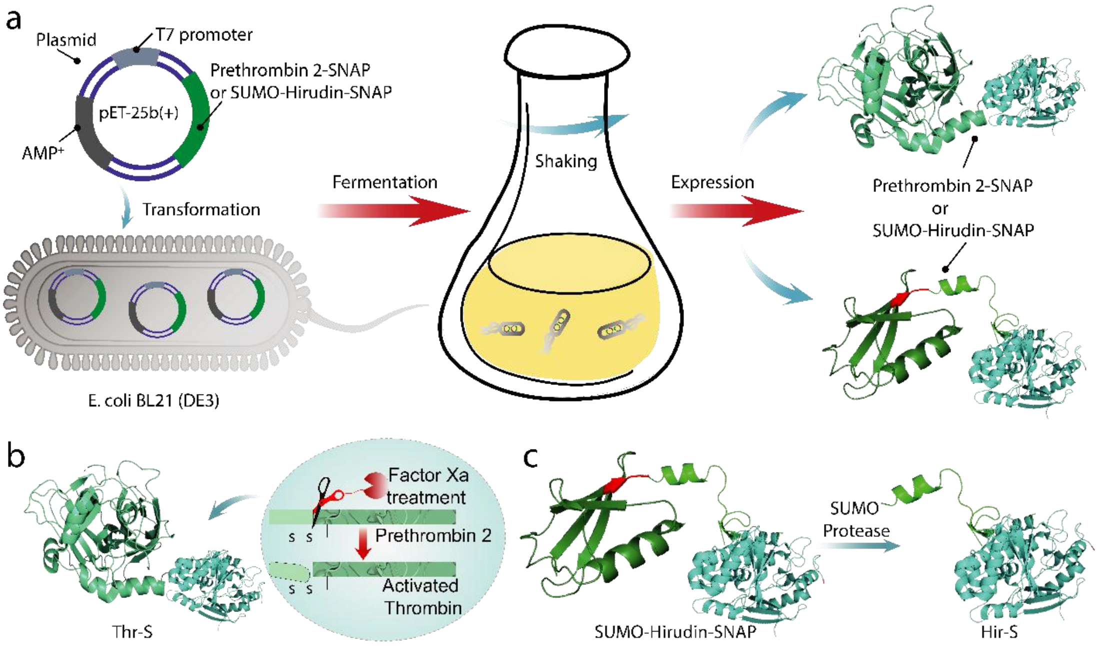

Supplementary Fig. 1. The vector design and expression of the recombinant fusion protein prothrombin 2-SNAP and SUMO-Hirudin-SNAP. (a) Plasmid design and expression of the recombinant prothrombin 2-SNAP and SUMO-Hirudin-SNAP. (b) Prethrombin 2 is the smallest single-chained immediate precursor of α-thrombin. After Factor Xa treatment, the active Thr-S was obtained. (c) SUMO tag was used for improving the expression of Hirudin, which could be removed using a SUMO protease during purification. Structures of Thr-S, SUMO-Hir-S, and Hir-S were extracted from the Protein Data Bank (PDB) and processed with PyMOL software. Thrombin/hirudin PDB ID: 4HTC; SUMO PDB ID: 1WM3; SNAP-tag PDB ID: 3KZY.

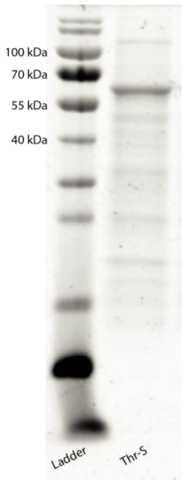

Supplementary Fig. 2. Coomassie-stained 15% SDS-PAGE image of recombinant Thr-S. Each

experiment was repeated three times independently with similar results.

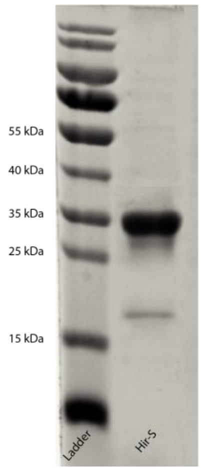

Supplementary Fig. 3. Coomassie-stained 15% SDS-PAGE image of recombinant Hir-S. Each experiment was repeated three times independently with similar results.

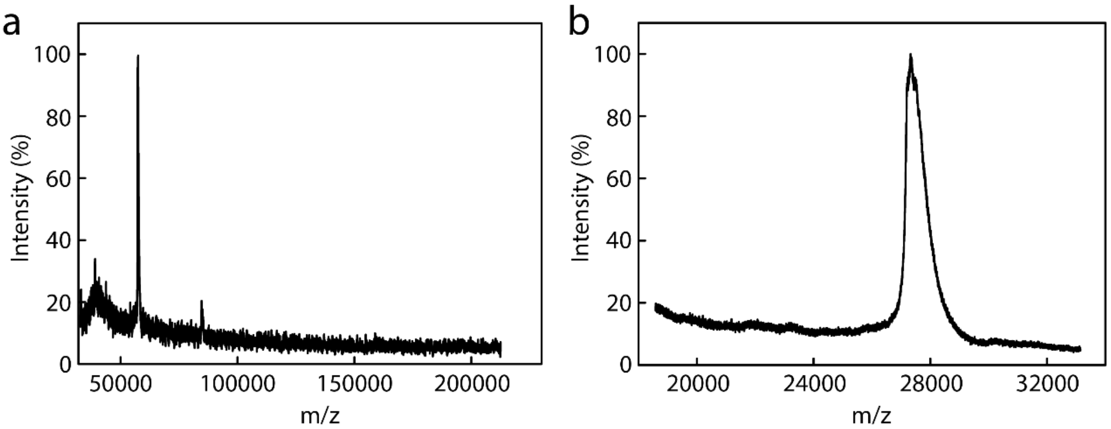

Supplementary Fig. 4. MALDI-TOF mass spectra of recombinant proteins. (a) Thr-S; (b) Hir-S. Each experiment was repeated three times independently with similar results.

Supplementary Table 1. Mass determination of proteins

	M calculated* (Da)	$M^{\#}$ (Da)
Thr-S	57103.3	57449.4±100
Hir-S	27339.8	27323.3±100

*average molar mass calculated with ExPASy ProtParam tool.

#molar mass determined by MALDI-TOF mass spectrometry.

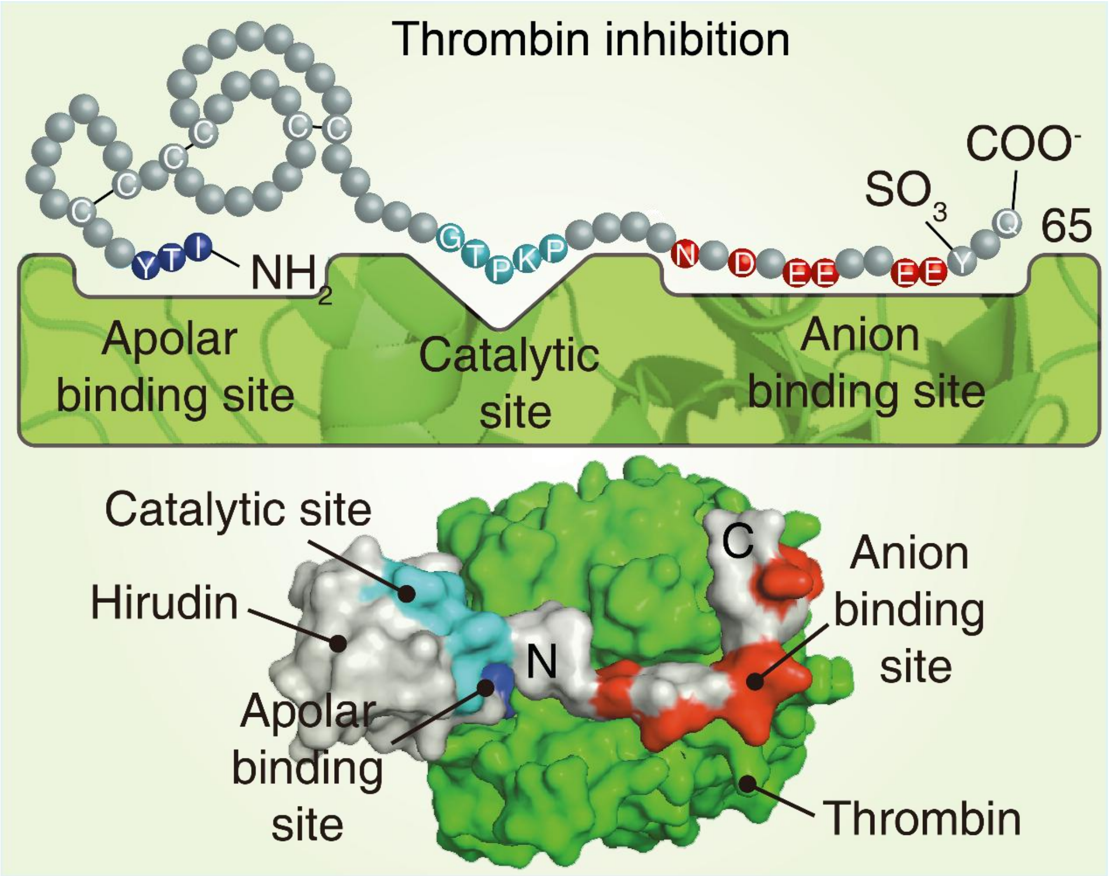

Supplementary Fig. 5. Schematic illustration of the binding mode of hirudin to thrombin. The key hydrophobic pocket binding residues are indicated in dark blue, and the anionic pocket binding residues are colored in red (PDB id 4HTC). The structural image was created using PyMOL (https://www.pymol.org/).

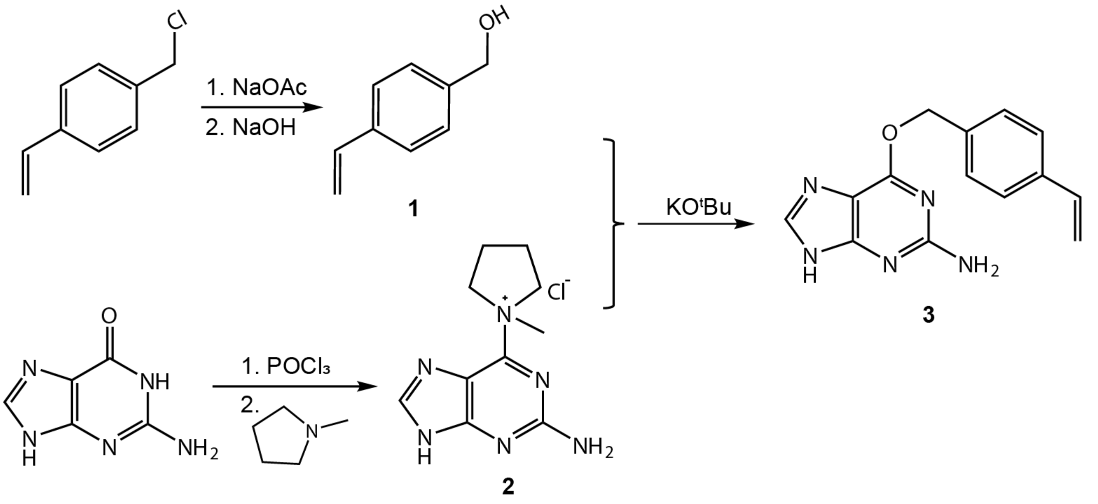

Supplementary Fig. 6. Synthesis scheme of the BS ligand.

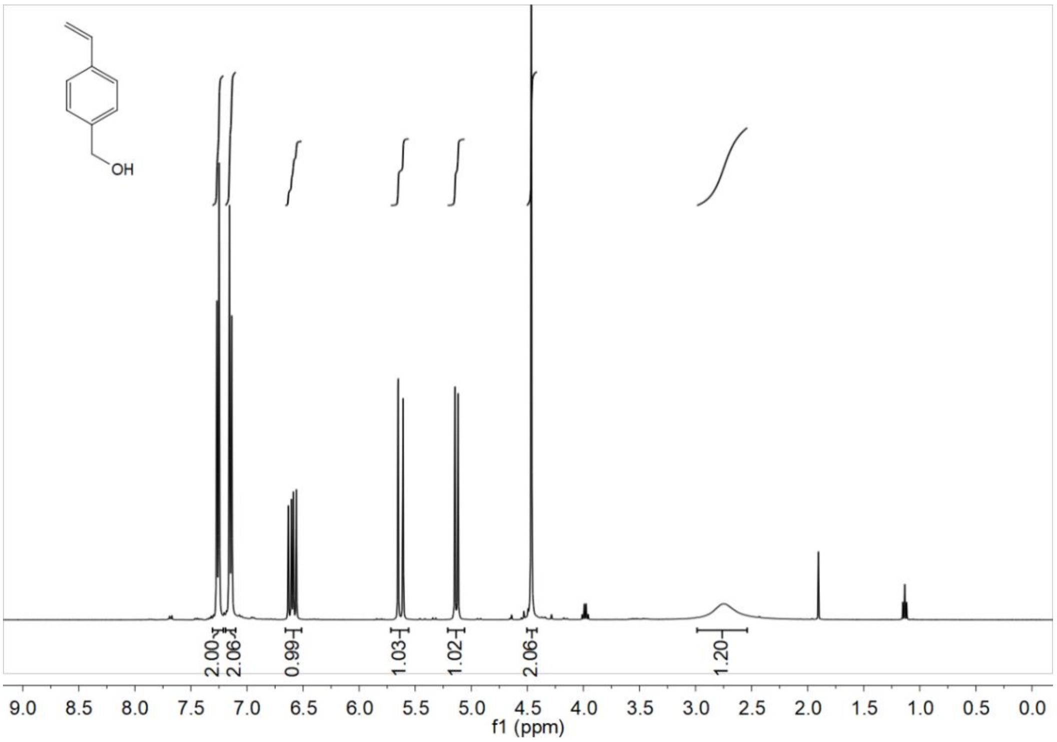

Supplementary Fig. 7. The ^{1}H-NMR spectrum of 4-vinylbenzyl alcohol (400 MHz, $CDCl_3$, r.t.) : δ=2.75 (s, 1H), 4.46 (s, 2H), 5.13 (d, J = 10.8 Hz, 1H), 5.63 (d, J = 17.6 Hz, 1H), 6.60 (m, 1H), 7.15 (d, J = 8 Hz, 2H), 7.26 (d, J = 8 Hz, 2H) ppm.

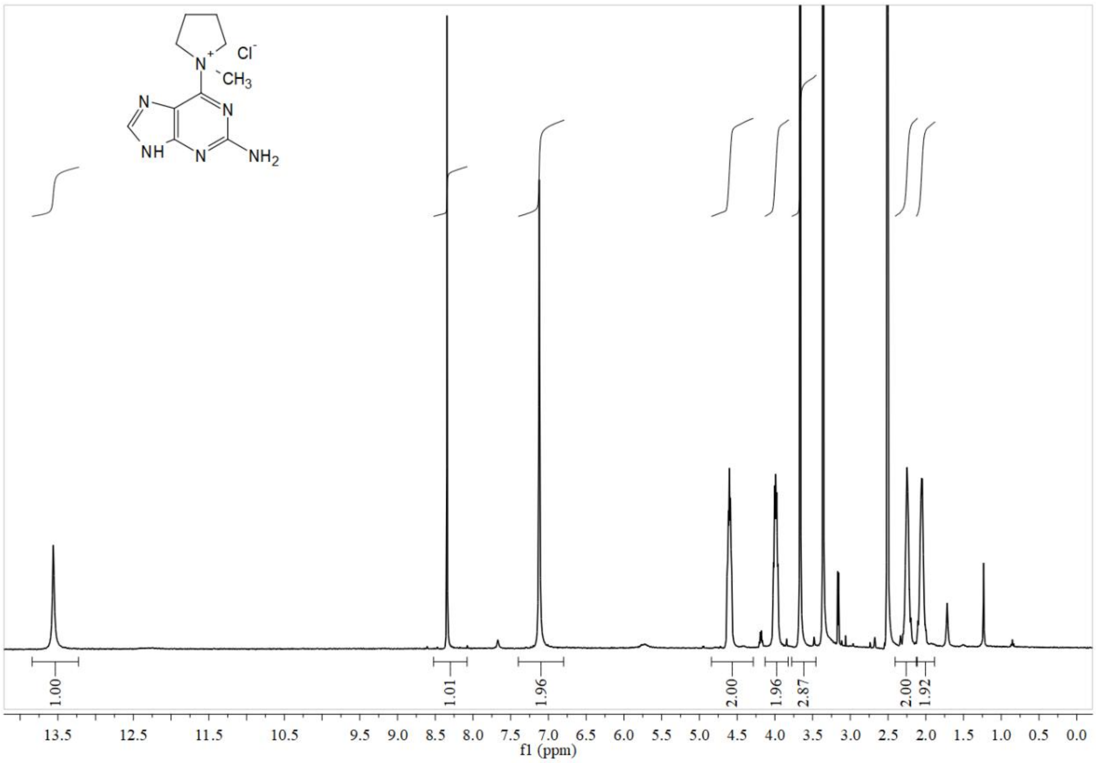

Supplementary Fig. 8. The ^{1}H-NMR spectrum of compound 2 (400 MHz, d6-DMSO, r.t.): δ=2.05 (m, 2H), 2.25 (m, 2H), 3.66 (s, 3H), 3.99 (m, 2H), 4.60 (m, 2H), 7.12 (s, 2H), 8.34 (s, 1H), 13.56 (s, 1H) ppm.

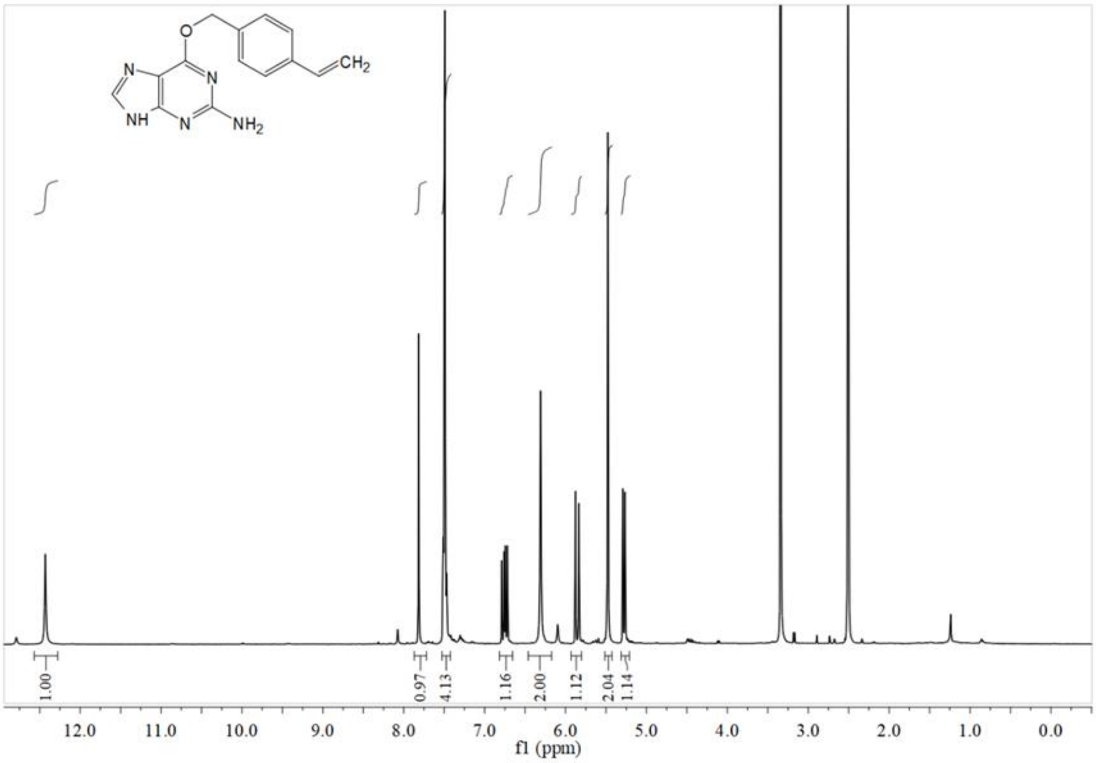

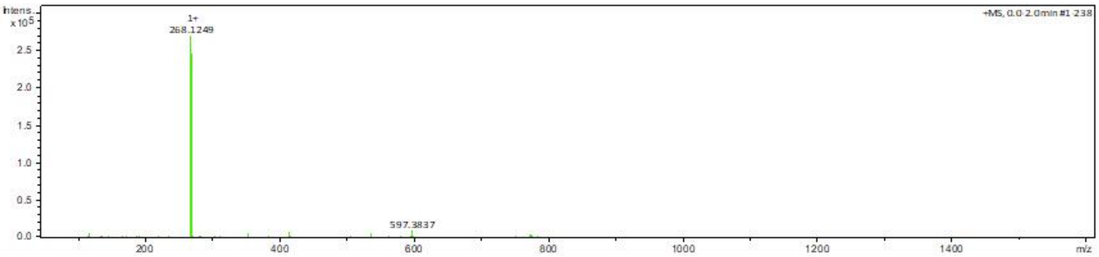

Supplementary Fig. 9. The ^{1}H-NMR spectrum (400 MHz, d6-DMSO, r.t.) : δ=5.28 (d, J = 10.92 Hz, 1H,), 5.48 (s, 2H), 5.86 (d, J = 17.68 Hz, 1H), 6.31 (s, 2H), 6.75 (m, 1H), 7.49 (d, J = 1.64 Hz, 4H), 7.82 (s, 1H), 12.43 (s, 1H) ppm. And ESI spectrum of the BS ligand.

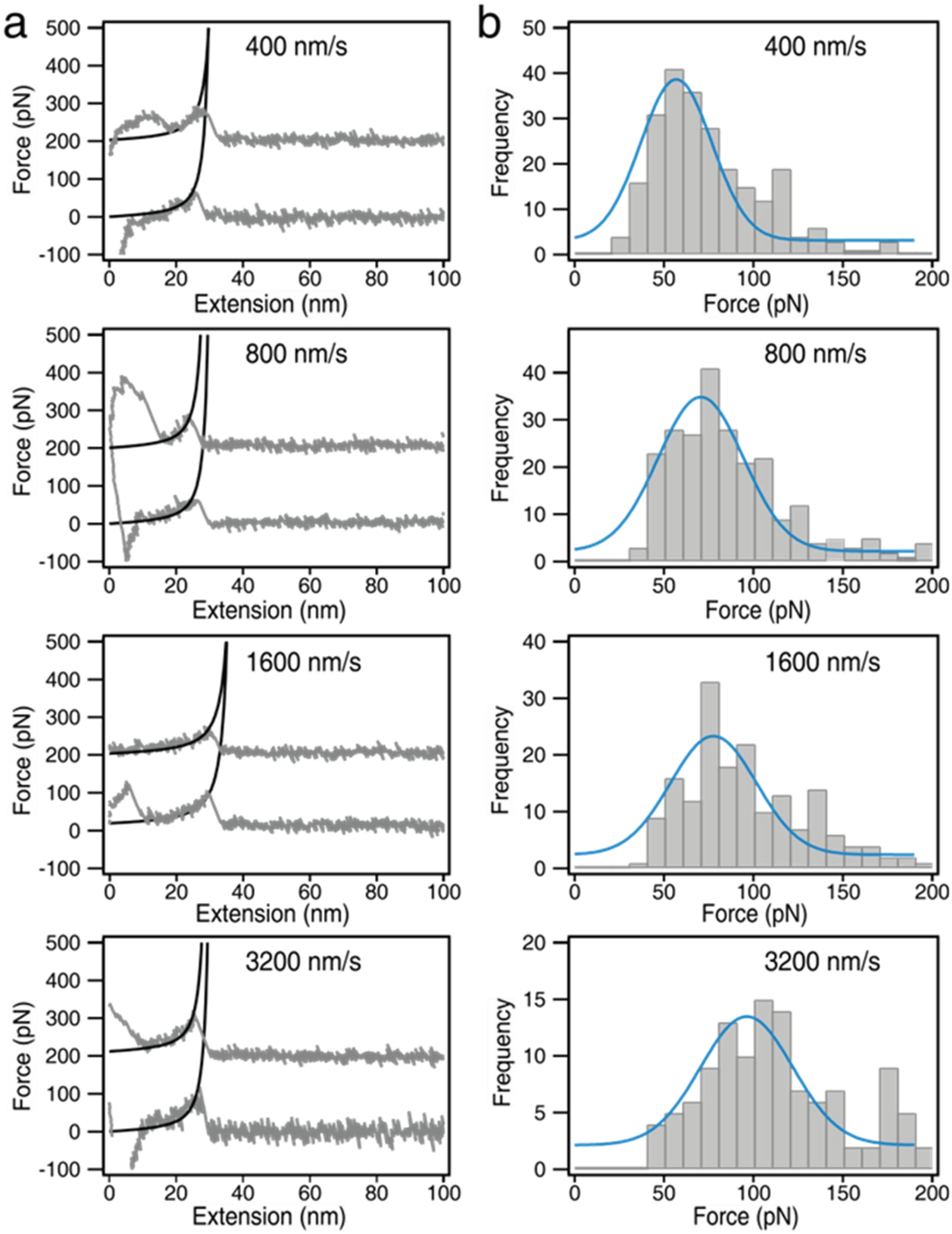

Supplementary Fig. 10. AFM-based single-molecule force measurements. (a) Typical force-retract curves for the rupture of the Thr-S and Hir-S complex at pulling speeds of 400, 800, 1600 and 3200 nm s^{-1}, respectively. Worm-like chain-fitting of the force-extension curves (black lines) confirmed that the peak at an extension range of 10-30 nm corresponds to the rupture of the Thr-S/Hir-S interaction. (b) Rupture force histograms at pulling speeds of 400, 800,1600 and 3200 nm s^{-1}, respectively. The Gaussian fitting shows the average rupture forces of 61.87 ± 19.87, 85.58 ± 23.82, 82.65 ± 24.00 and 101.25 ± 25.78 pN, respectively. Each experiment was repeated three times independently with similar results. Data are presented as mean values with error bars representing the standard deviation of three independent replicates (n = 3 in each group).

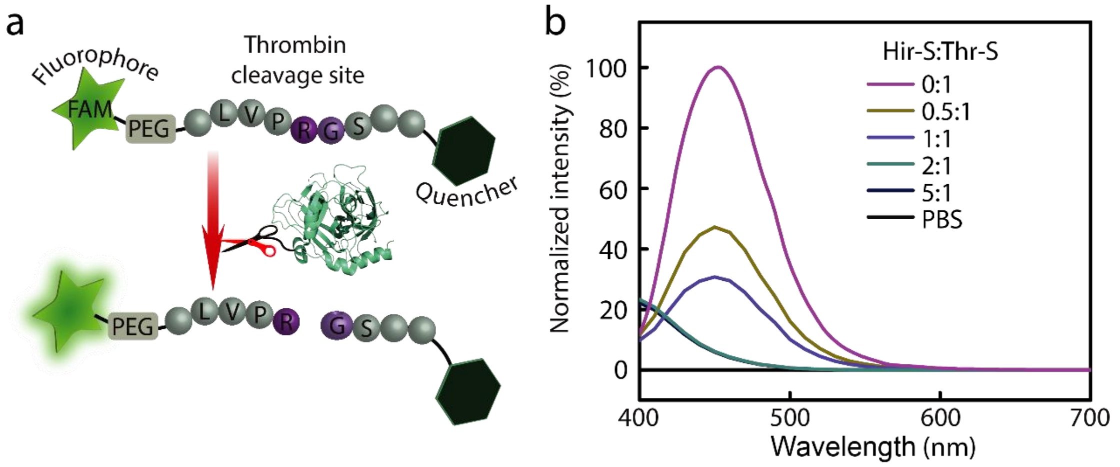

Supplementary Fig. 11. (a) Schematic illustration of the cleavage of the fluorogenic substrate producing a 5(6)-carboxyfluorescein-bearing peptide fragment with strong fluorescence at 450 nm. (b) Determination of the best ratio of thrombin and hirudin for complete thrombin inhibition. Each experiment was repeated three times independently with similar results.

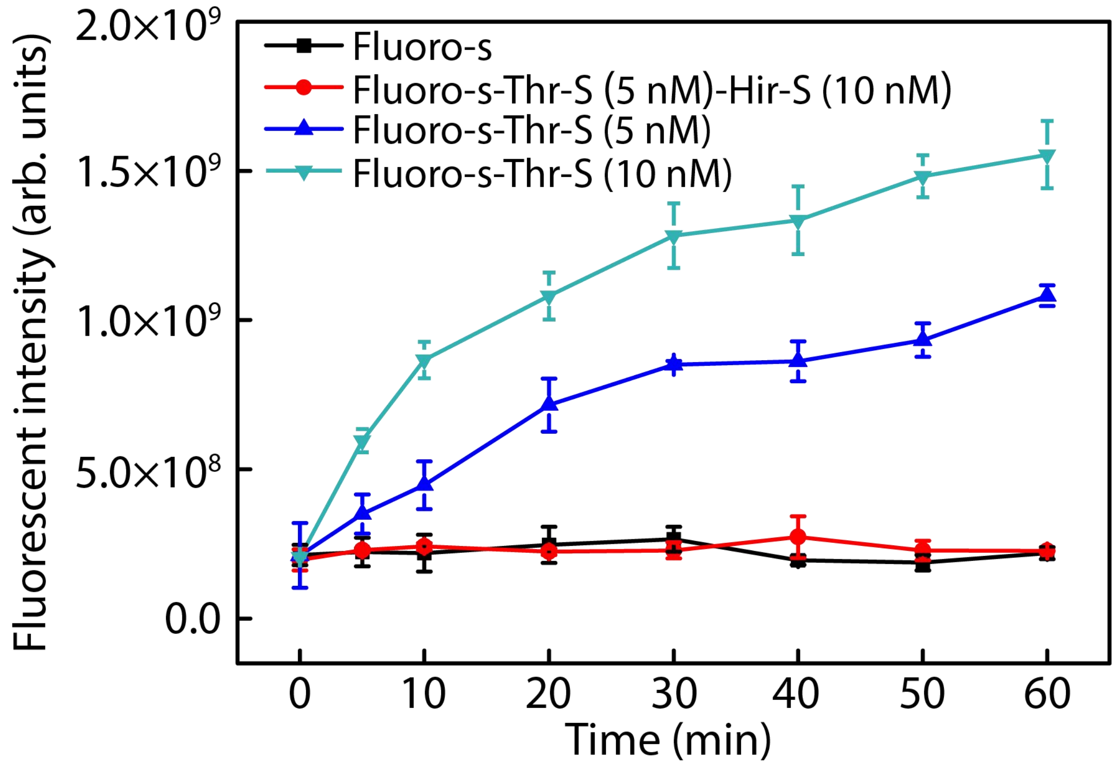

Supplementary Fig. 12. Evaluation of the activity of Thr-S in the presence or absence of Hir-S in solution using a fluorogenic substrate. The fluorescence intensity was recorded as the function of time at 450 nm. Four groups (pure Fluoro-s, Fluoro-s-Thr-S (5 nM)-Hir-S (10 nM), Fluoro-s-Thr-S (5 nM) and Fluoro-s-Thr-S (10 nM)) were measured to verify the enzyme activity. The mixtures were incubated in a 96-well plate at room temperature for 60 min, and the fluorescence was measured to monitor the reaction kinetics. Each experiment was repeated three times independently with similar results. Data are presented as mean values with error bars representing the standard deviation of three independent replicates (n = 3 in each group).

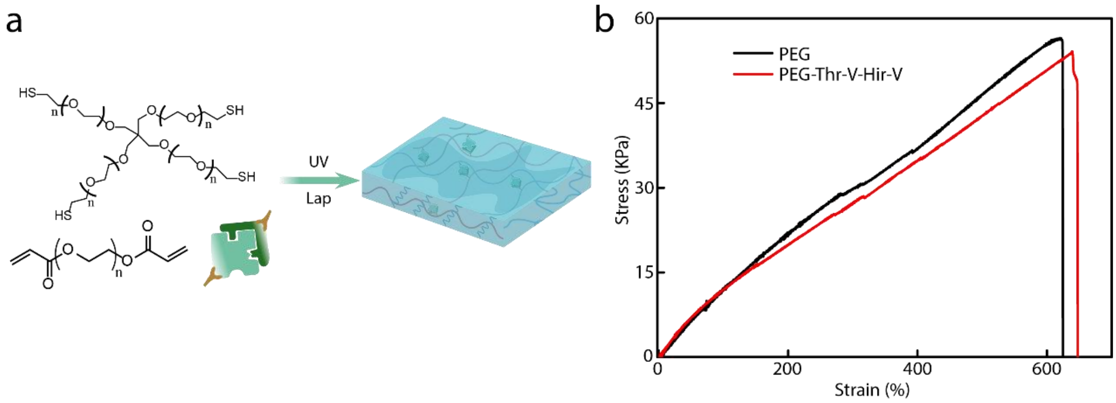

Supplementary Fig. 13. Hydrogel preparation and mechanical properties. (a) The hydrogel precursor mixture is composed of 4-arm-PEG-SH, PEGDA, LAP (photo initiator) and thrombin-hirudin pairs. The gelation process is activated by UV irradiation for 20 min. (b) Typical stress-strain curves of the developed hydrogels with or without the enzyme. Each experiment was repeated three times independently with similar results.

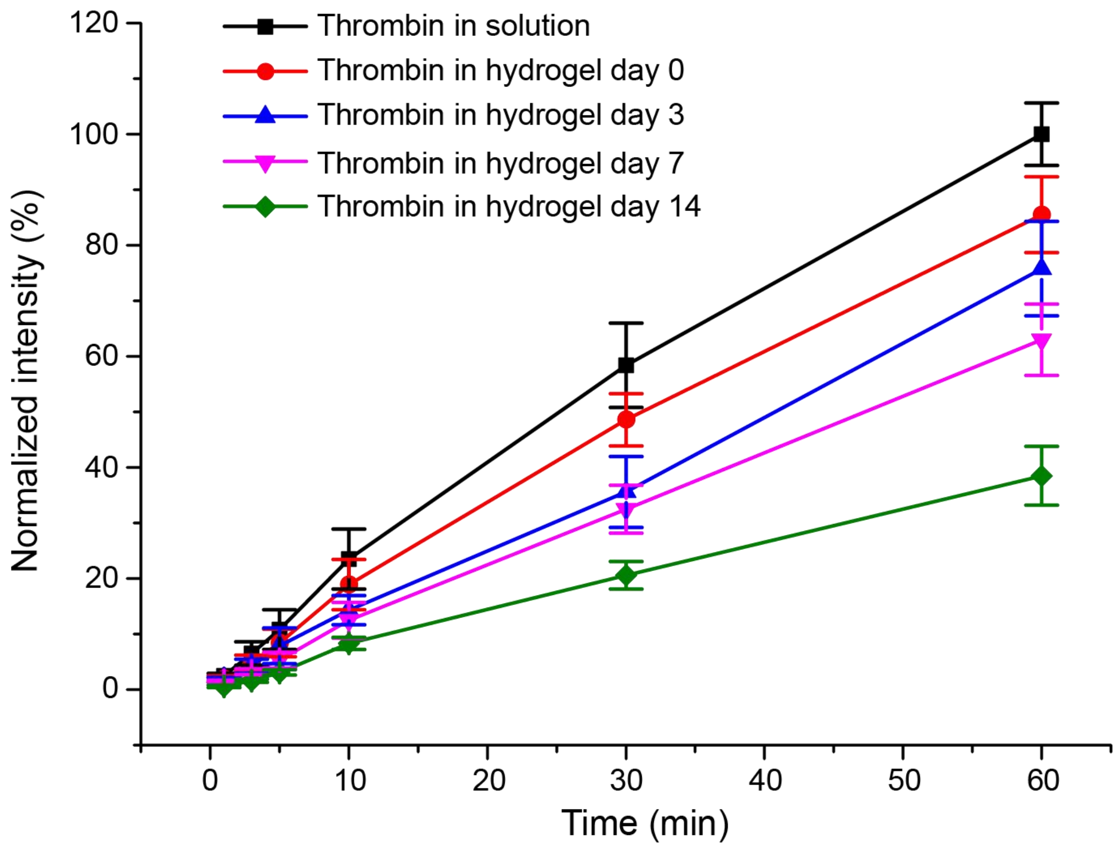

Supplementary Fig. 14. Measurements of enzyme activity in solution and hydrogels that were stored at 4 °C for 0, 3, 7 and 14 days.

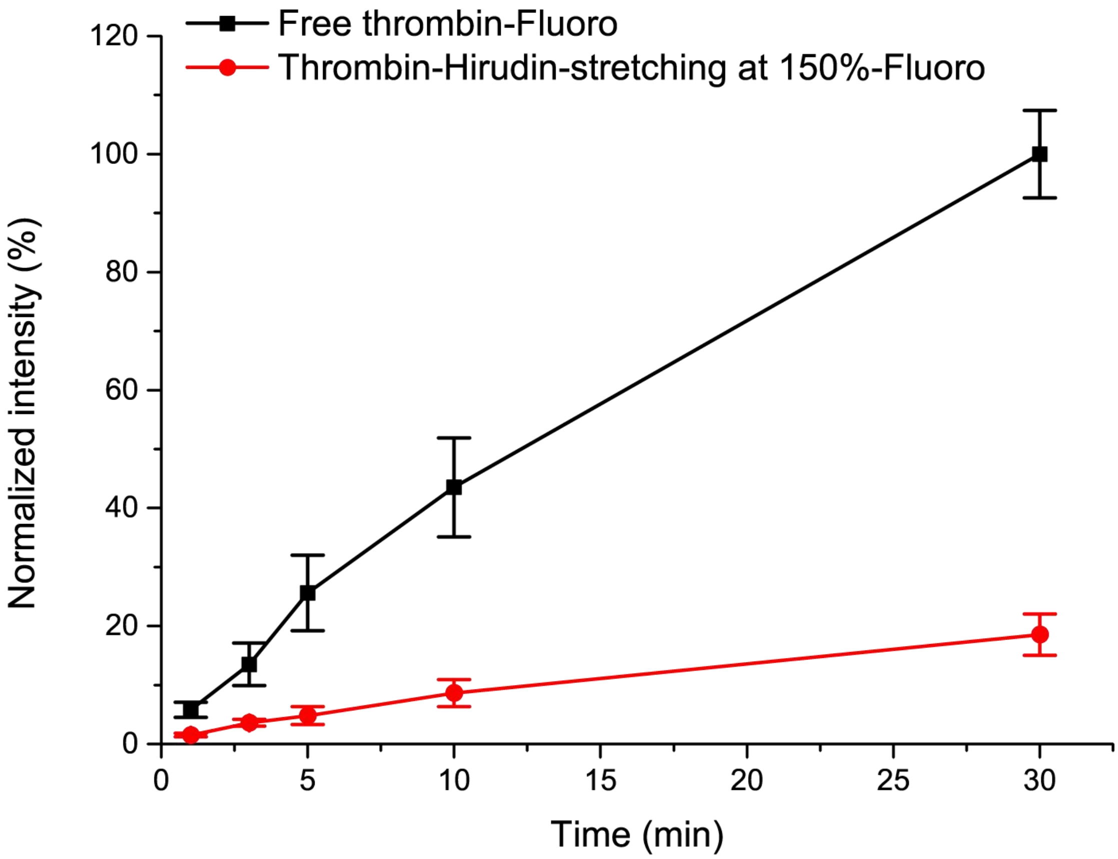

Supplementary Fig. 15. The evaluation of dissociation ratio of thrombin-hirudin complex at a strain of 150%.

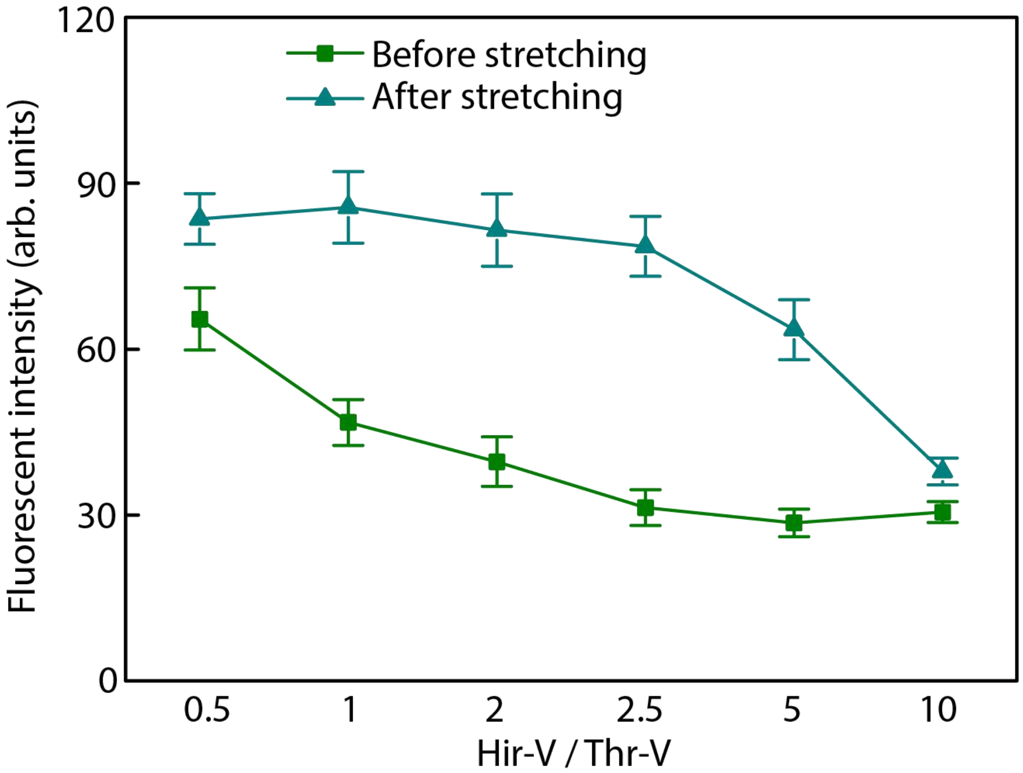

Supplementary Fig. 16. The influence of the ratio of Hir-S and Thr-S for the activation of thrombin under strain of 150% measured with a fluoregenic substrate. Each experiment was repeated three times independently with similar results. Data are presented as mean values with error bars representing the standard deviation of three independent replicates (n = 3 in each group).

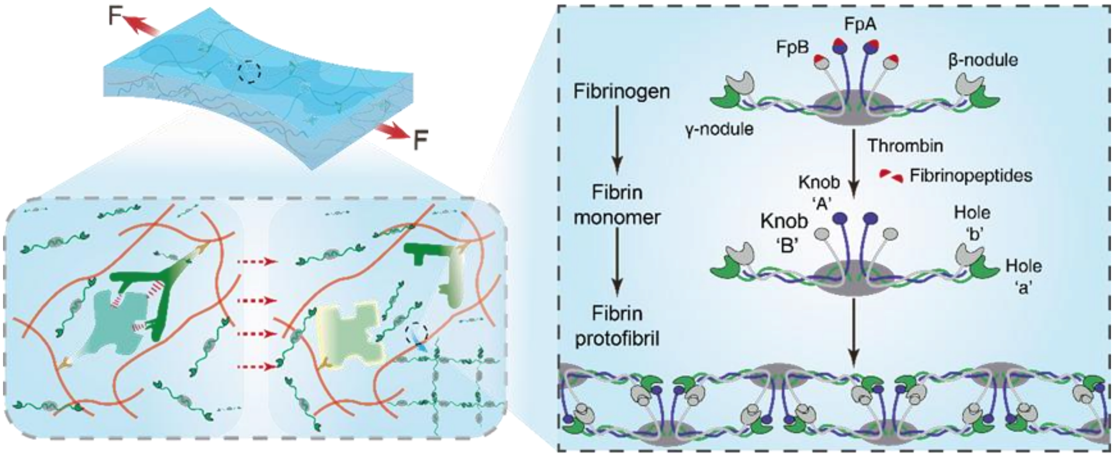

Supplementary Fig. 17. Illustration of the formation of a new network inside the hydrogels triggered by the mechanically activated enzyme thrombin (left). The mechanism of the transformation of fibrinogen into fibrin fibers induced by thrombin (right).

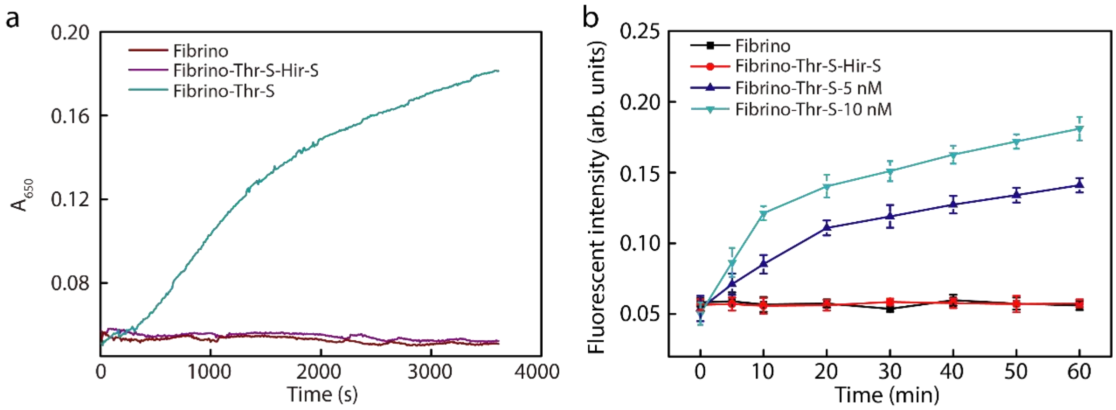

Supplementary Fig. 18. Evaluation of the activity of Thr-S in the presence or absence of Hir-S in solution using fibrinogen as a substrate. (a) The absorbance increased at 650 nm resulting from the transformation of soluble fibrinogen to insoluble fibrin fibers. (b) The time-dependent absorbance based on the conversion of fibrinogen to fibrin fibers. Briefly, Thr-S (100/200 nM) was added into 180 μl of PBS buffer and 10 μl of fibrinogen (10 mg/mL) was added into the mixture. The absorbance intensity was monitored using a microplate reader spectrophotometer every 1 min. Each experiment was repeated three times independently with similar results. Data are presented as mean values with error bars representing the standard deviation of three independent replicates (n = 3 in each group).

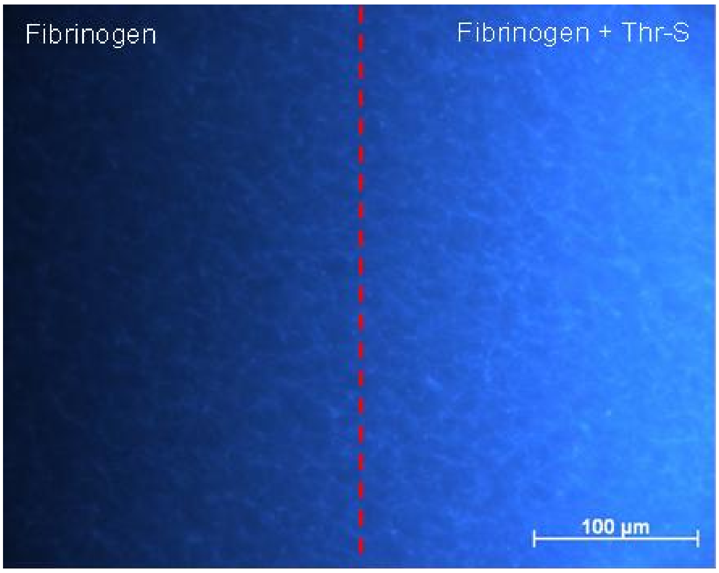

Supplementary Fig. 19. Dark field picture of fibrinogen treated with thrombin. Thr-S was added from the right side of the fibrinogen solution. It was observed that the field of view brightened from right side to left side, indicating the conversion of fibrinogen into fibrin fibers. Each experiment was repeated three times independently with similar results.

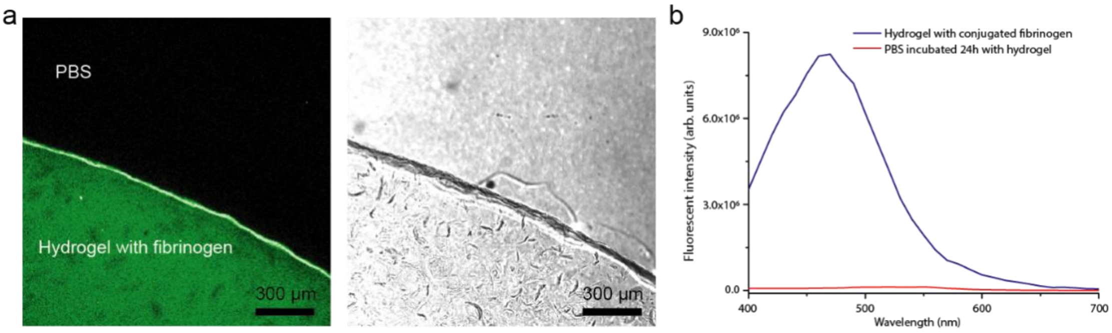

Supplementary Fig. 20. Examination of fibrinogen state in hydrogels. (a). Confocal images for fluoro-labeled fibrinogen in hydrogels, left: bright field, right: dark field; (b). Fluorescent spectrum of fibrinogen contained hydrogels and PBS solution incubated with hydrogel for 24 hours.

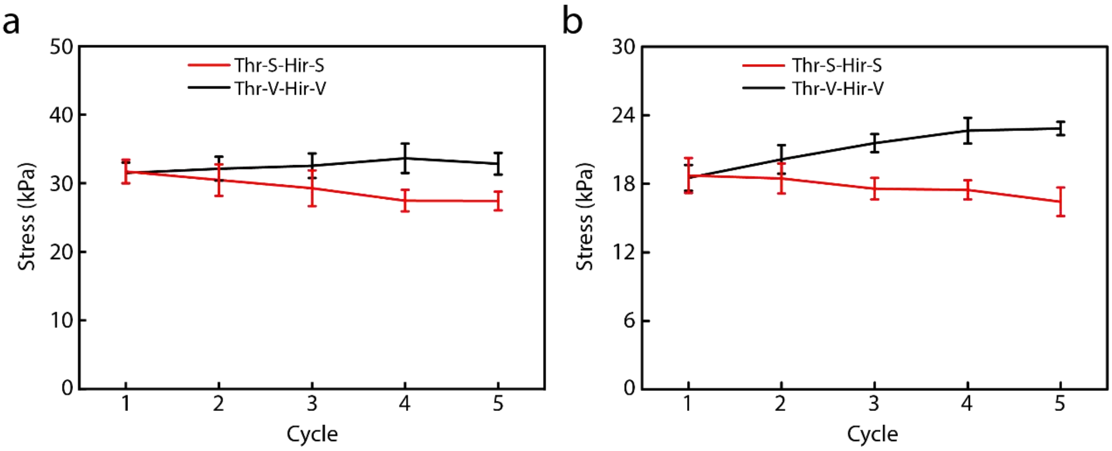

Supplementary Fig. 21. Enzyme triggered self-stiffening hydrogels with applied strain of (a) 150% and (b) 300%. Each experiment was repeated three times independently with similar results. Data are presented as mean values with error bars representing the standard deviation of three independent replicates (n = 3 in each group).

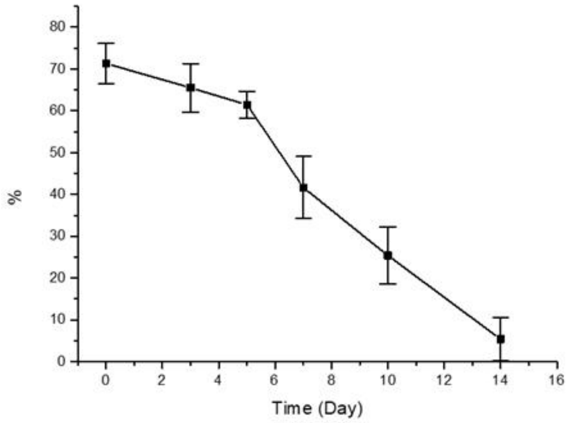

Supplementary Fig. 22. The evaluation of stability of fibrinogen in hydrogel through mechano-triggered self- stiffening property after storing for 14 days.

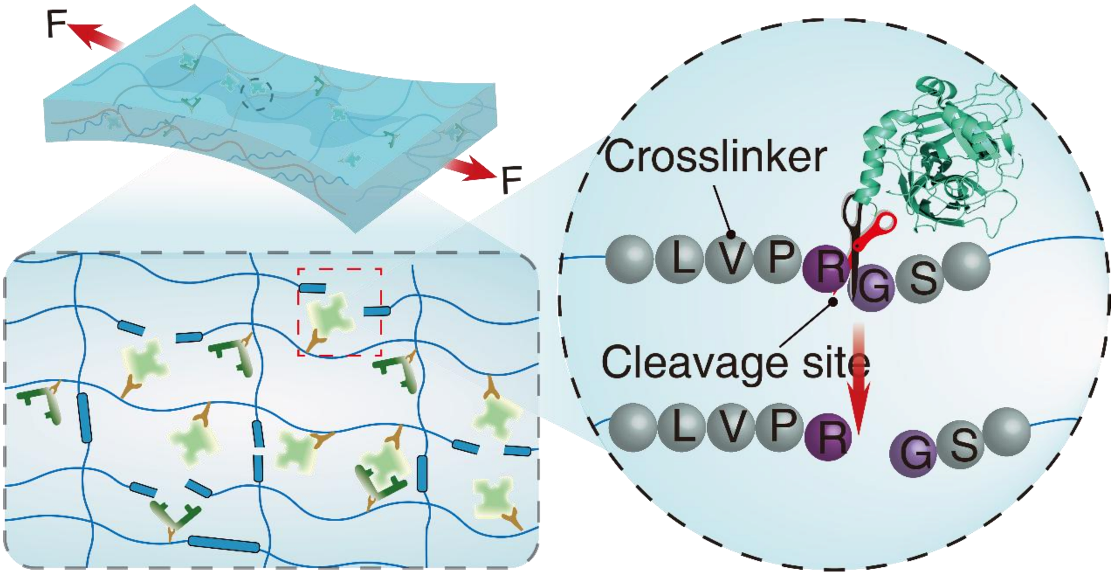

Supplementary Fig. 23. Schematic illustration of mechano-triggered peptide hydrolysis occurring within self-softening hydrogels. The peptide crosslinkers of the hydrogels are cleaved by mechano-activated thrombin.

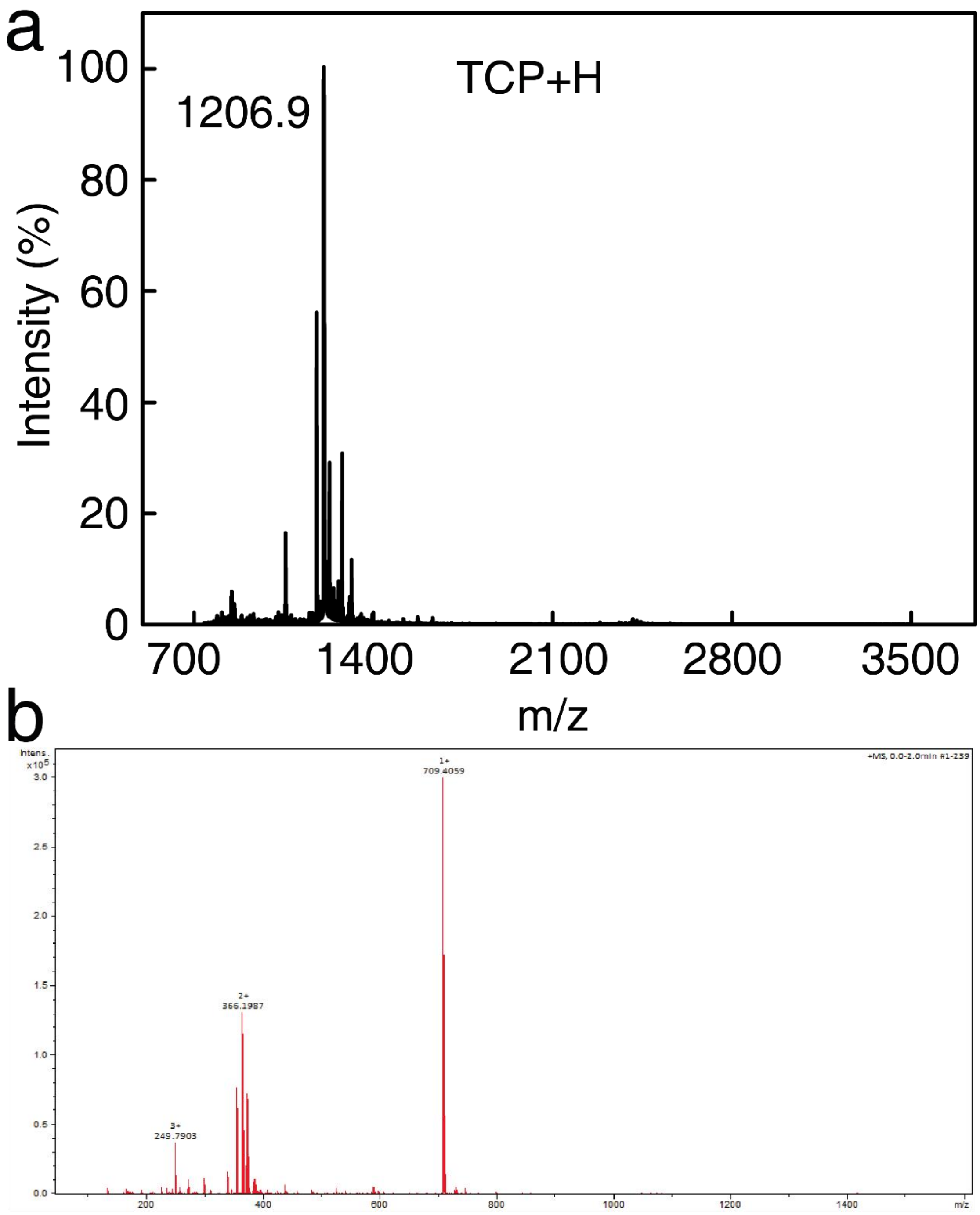

Supplementary Fig. 24. (a) MALDI-TOF mass spectrum of TCP crosslinker. (b) ESI spectrum of TCP crosslinker after Thr-S treatment. TCP was completely digested by Thr-S. Each experiment was repeated three times independently with similar results.

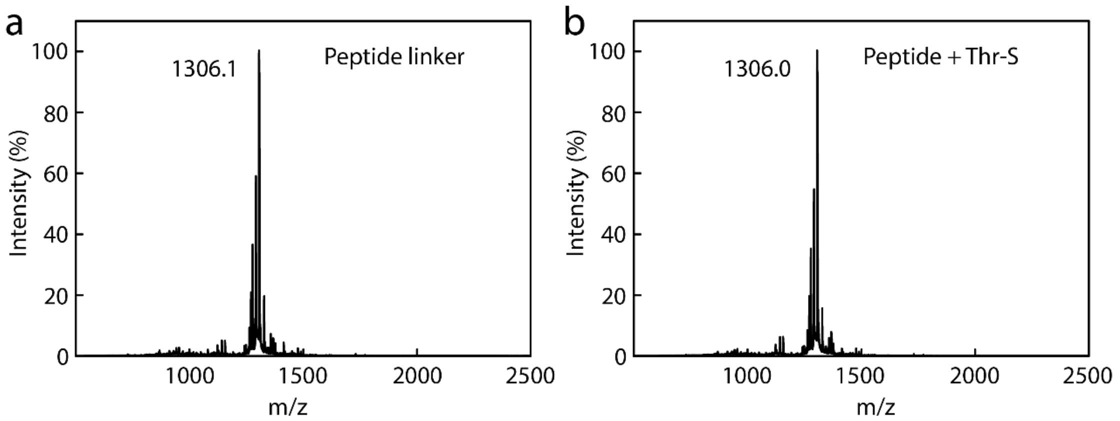

Supplementary Fig. 25. MALDI-TOF mass spectra of thrombin non-cleavable peptide crosslinker before (a) and after (b) Thr-S treatment.

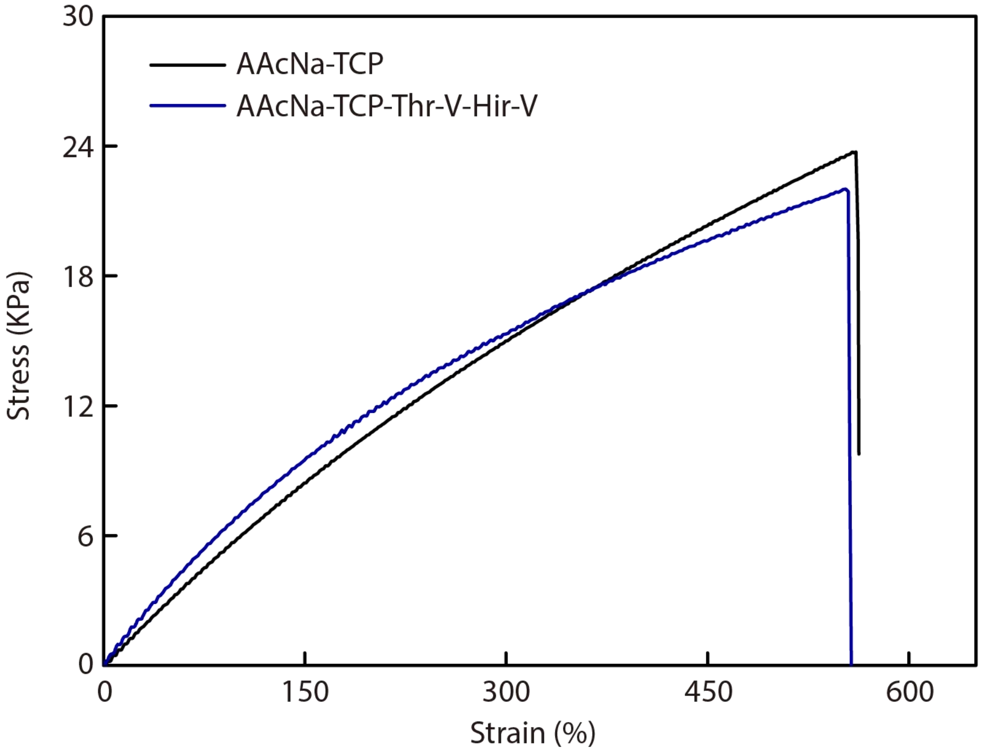

Supplementary Fig. 26. Mechanical property of the AAcNa based hydrogels. Each experiment was repeated three times independently with similar results.

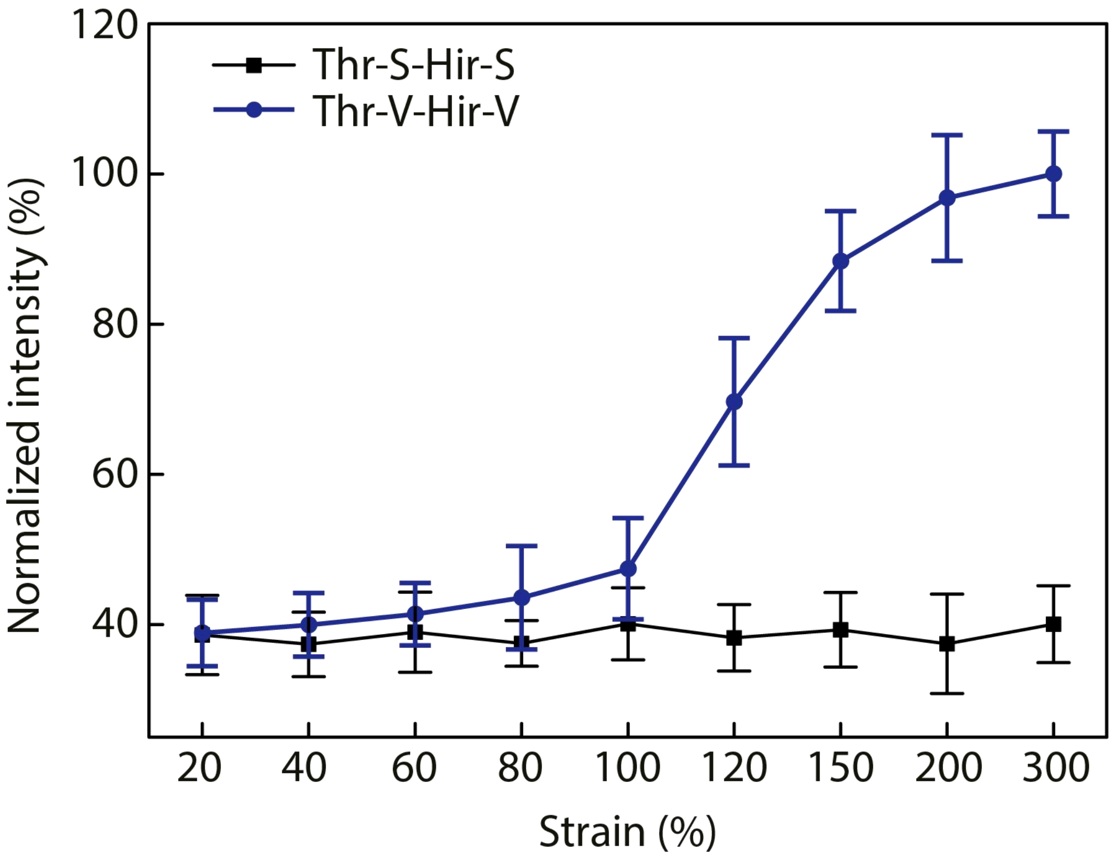

Supplementary Fig. 27. The evolution of fluorescence intensity of AAcNa hydrogels after applying stretching strains from 20% to 300%. Each experiment was repeated three times independently with similar results. Data are presented as mean values with error bars representing the standard deviation of three independent replicates (n = 3 in each group).

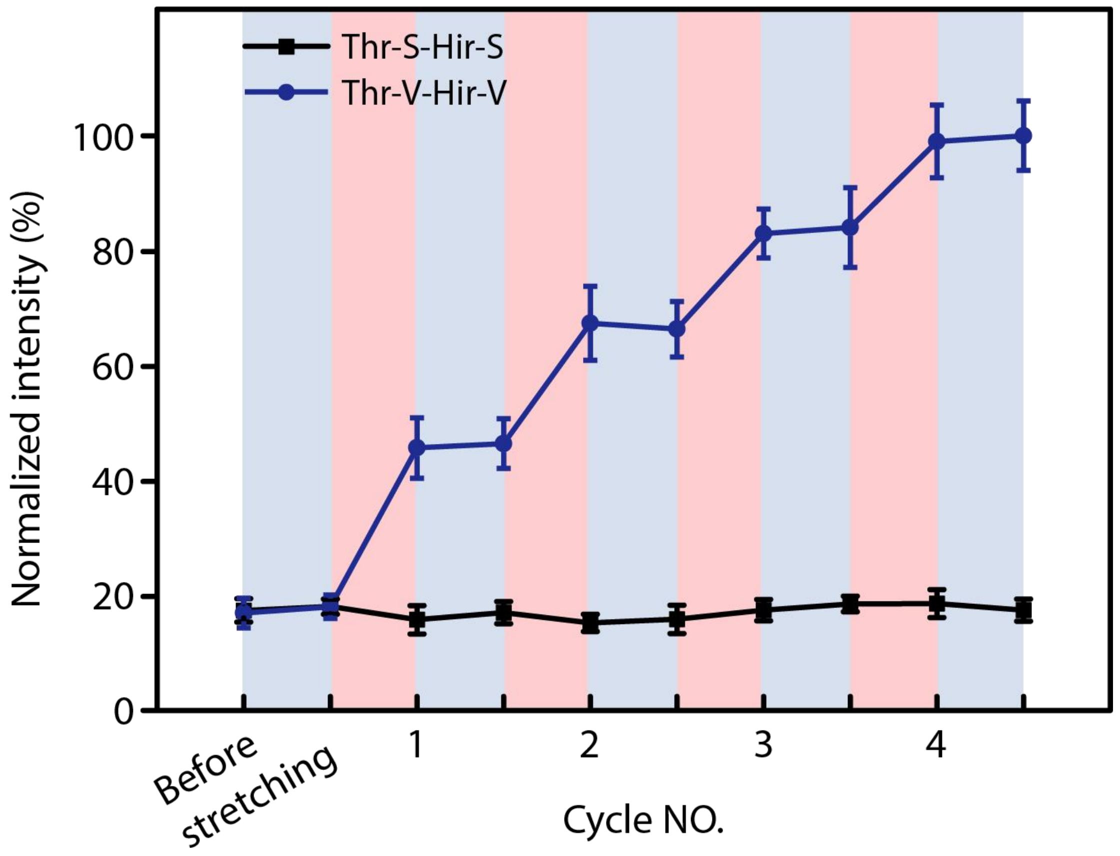

Supplementary Fig. 28. Controlled biocatalytic activation through stretch/release cycles on AAcNa hydrogels measured by cleavage of a fluoregenic substrate. Each experiment was repeated three times independently with similar results. Data are presented as mean values with error bars representing the standard deviation of three independent replicates (n = 3 in each group).

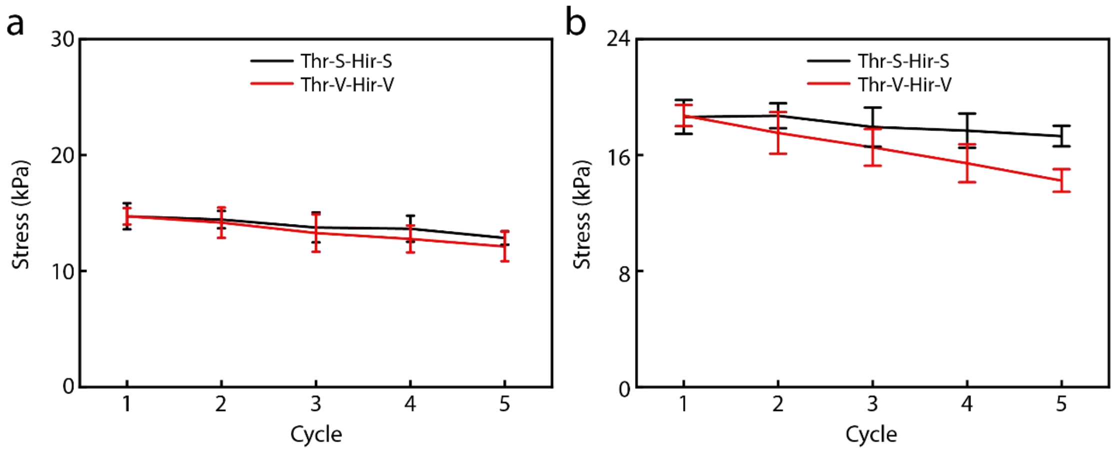

Supplementary Fig. 29. Enzyme triggered self-stiffening hydrogels with strains of (a) 300% and (b) 450%. Each experiment was repeated three times independently with similar results. Data are presented as mean values with error bars representing the standard deviation of three independent replicates (n = 3 in each group).

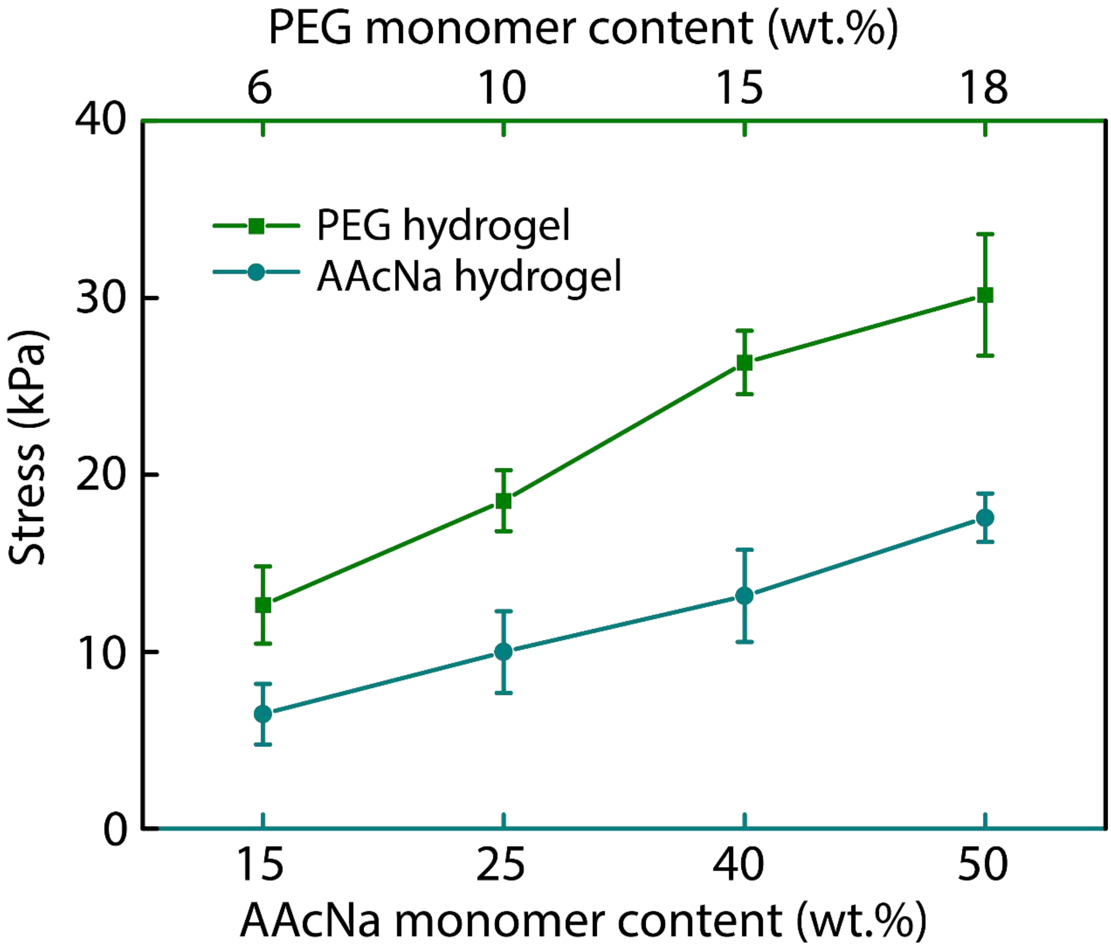

Supplementary Fig. 30. Mechanical properties of PEG and AAcNa hydrogels of different weight percentage (wt. %) under a strain of 300%. According to these results, PEG hydrogels with 6 wt.% and AACNa hydrogels with 40 wt.%. were chosen for the preparation of bilayered shape-morphing hydrogels. All experiments were repeated as triplicates and error bars represent standard deviations. Each experiment was repeated three times independently with similar results. Data are presented as mean values with error bars representing the standard deviation of three independent replicates (n = 3 in each group).

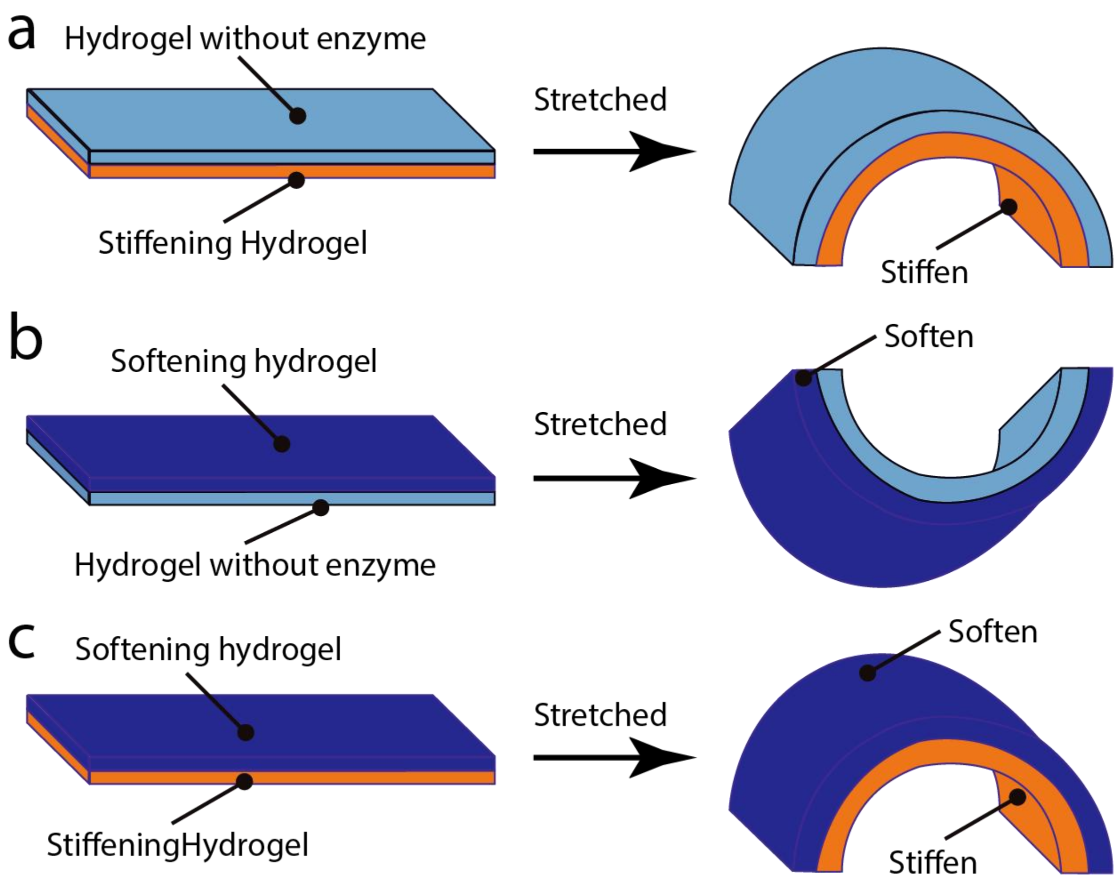

Supplementary Fig. 31. Schematic illustration of mechanically triggered shape-morphing hydrogels consisting of bilayer polymer networks. The bilayered hydrogels bend to the part with higher mechanical properties.

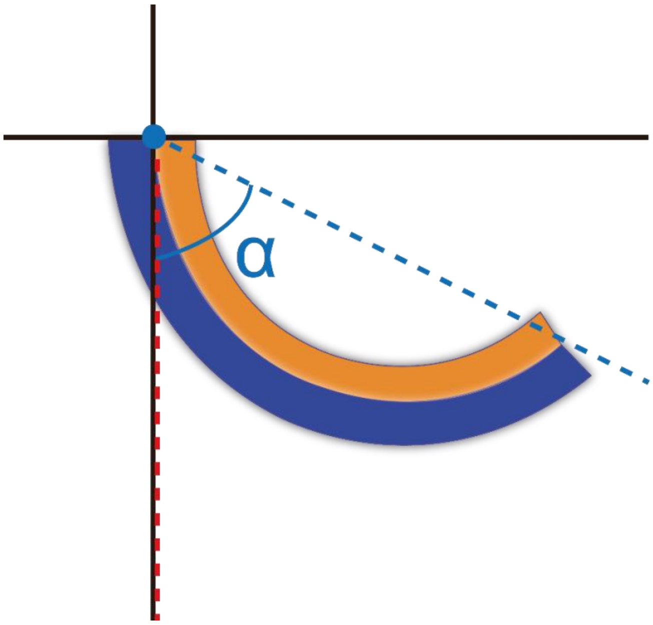

Supplementary Fig. 32. Schematic illustration of the measurement of the bending angle of the bilayered hydrogels. It is defined as the degree of deviation (α) from the original linear position (the red dashed line).

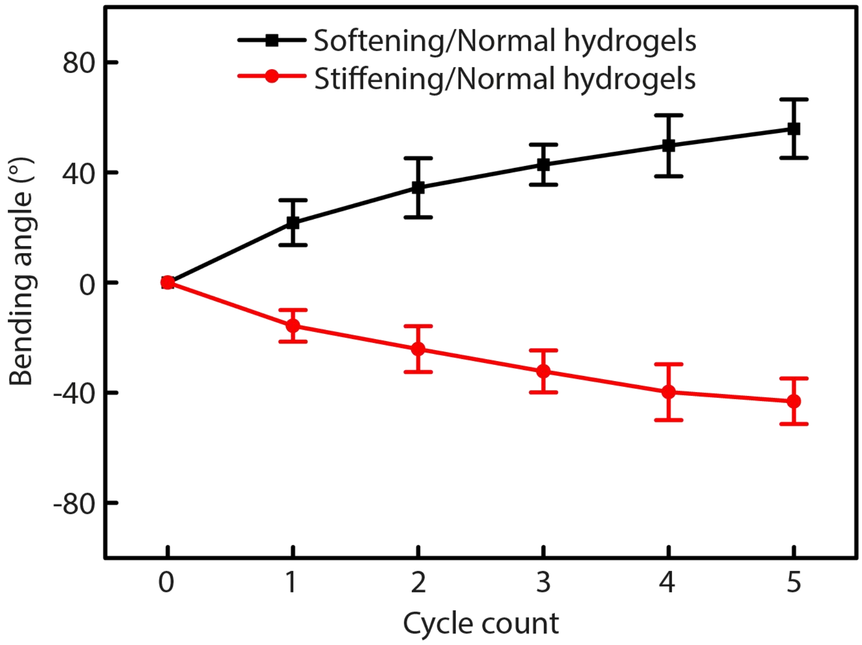

Supplementary Fig. 33. The evolution of bending angles of bilayer hydrogels from which only one-layer is mechanically actuatable under different tensile cycles regulated by enzyme activity (self-softening or self-stiffening). Each experiment was repeated three times independently with similar results. Data are presented as mean values with error bars representing the standard deviation of three independent replicates (n = 3 in each group).

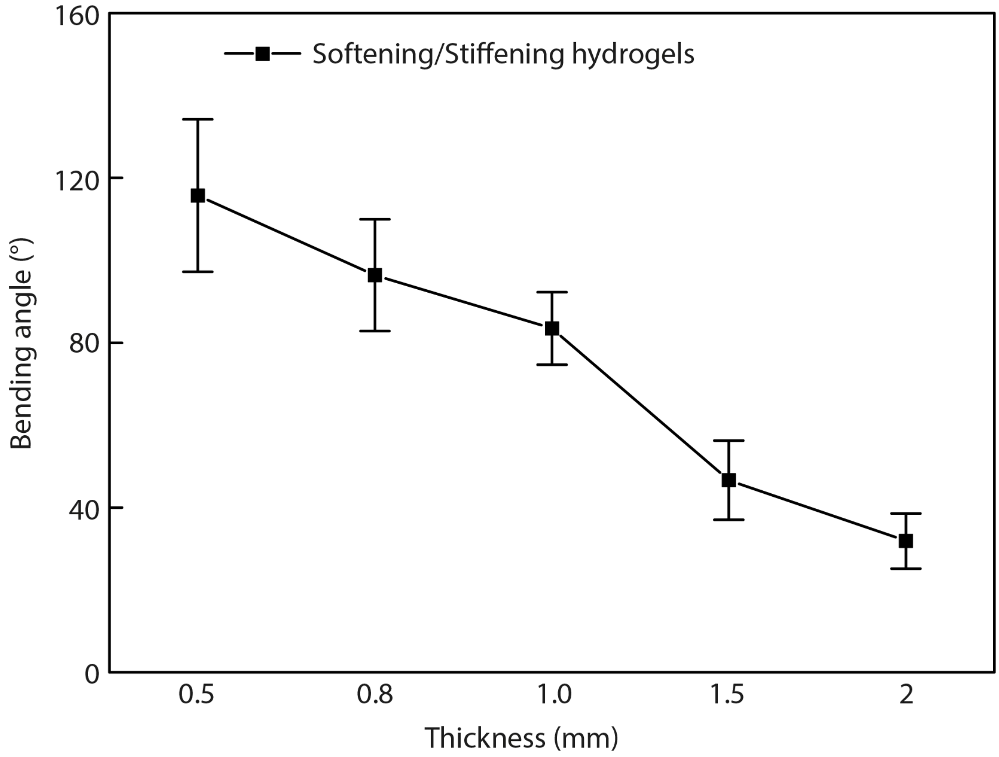

Supplementary Fig. 34. Evolution of bending angles of bilayered hydrogel as a function of the hydrogel thickness. All experiments were repeated as triplicates and error bars represent standard deviations. Each experiment was repeated three times independently with similar results. Data are presented as mean values with error bars representing the standard deviation of three independent replicates (n = 3 in each group).

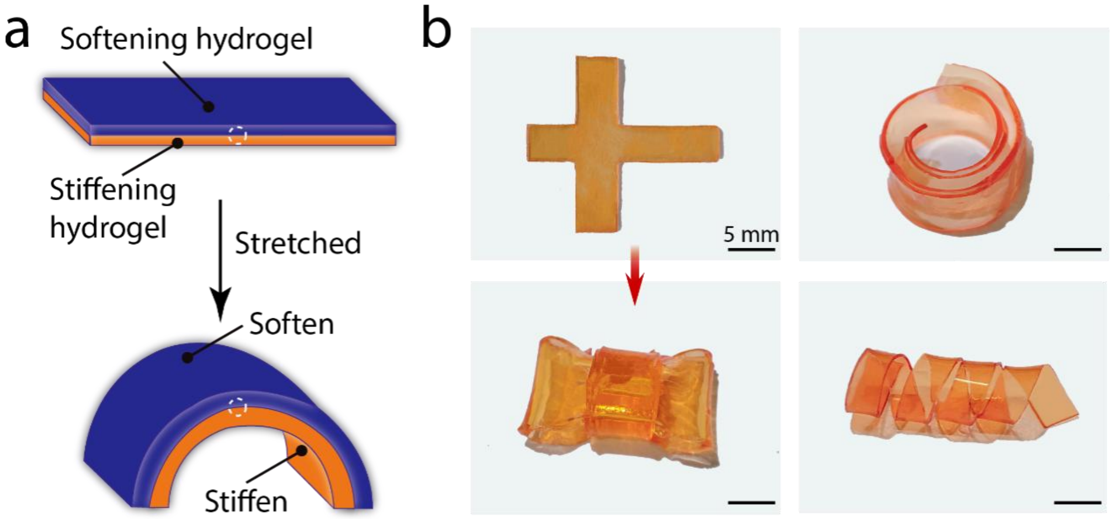

Supplementary Fig. 35. (a) Schematic illustration of mechano-triggered shape-morphing behavior of bilayered hydrogels. (b) Mechanically programmed 2D to 3D shape transformation of the bilayered hydrogels to realize a bowknot shape and helix shapes. Scale bar: 5 mm. Each experiment was repeated three times independently with similar results.

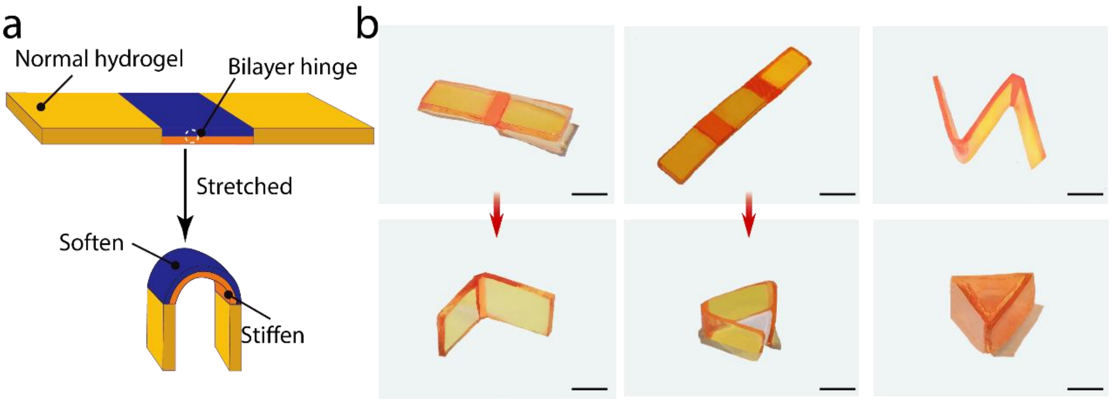

Supplementary Fig. 36. (a) Schematic illustration of the transformation of the bilayered hydrogels containing hinges that shape morph under mechanical stretching. (b) Pictures of the programmed 2D hydrogel structures transformed into distinct 3D architectures. Scale bar: 5 mm. Each experiment was repeated three times independently with similar results.

Supplementary References

1 Jackson, A. W., Stakes, C. & Fulton, D. A. The formation of core cross-linked star polymer and nanogel assemblies facilitated by the formation of dynamic covalent imine bonds. *Polym. Chem.* **2**, 2500-2511 (2011).

2 Lei, H. *et al.* Stretchable hydrogels with low hysteresis and anti-fatigue fracture based on polyprotein cross-linkers. *Nat. Commun.* **11**, 4032 (2020).

3 Keppler, A. *et al.* A general method for the covalent labeling of fusion proteins with small molecules in vivo. *Nat. Biotechnol.* **21**, 86-89 (2003).

4 Kobayashi, T. *et al.* Highly activatable and environment-insensitive optical highlighters for selective spatiotemporal imaging of target proteins. *J. Am. Chem. Soc.* **134**, 11153-11160 (2012).
