# Supplementary figures and images for: Shape morphing of hydrogels by harnessing enzyme enabled mechanoresponse
Source: Nat Commun. 2024 Jan 4;15:249. doi: 10.1038/s41467-023-44607-y (PMC10764310; doi:10.1038/s41467-023-44607-y)

Refer to Supplementary Fig. 2


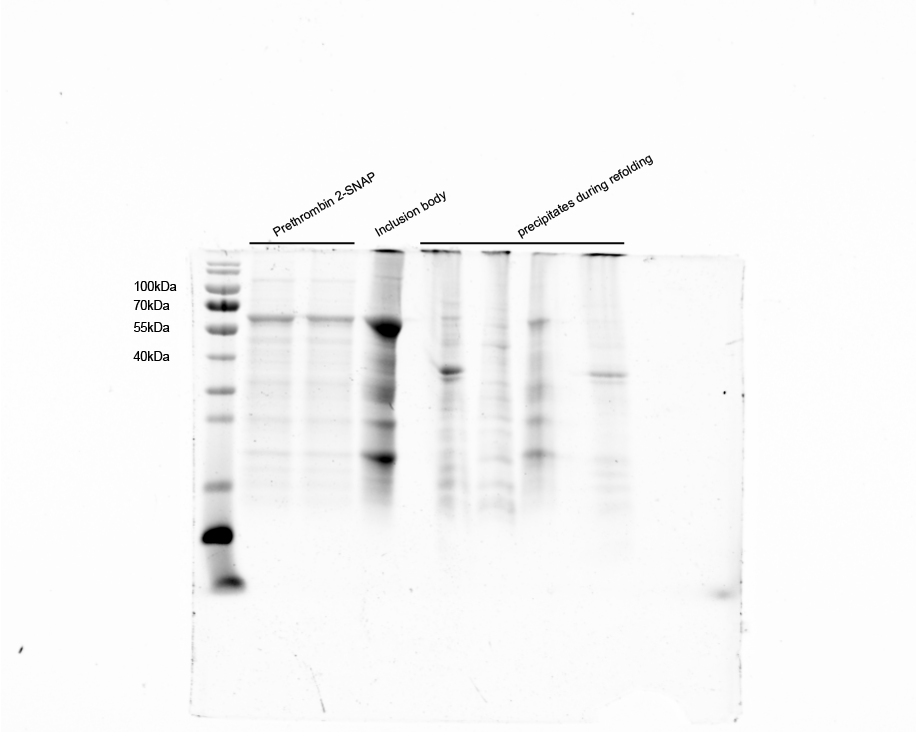


Refer to Supplementary Fig. 3


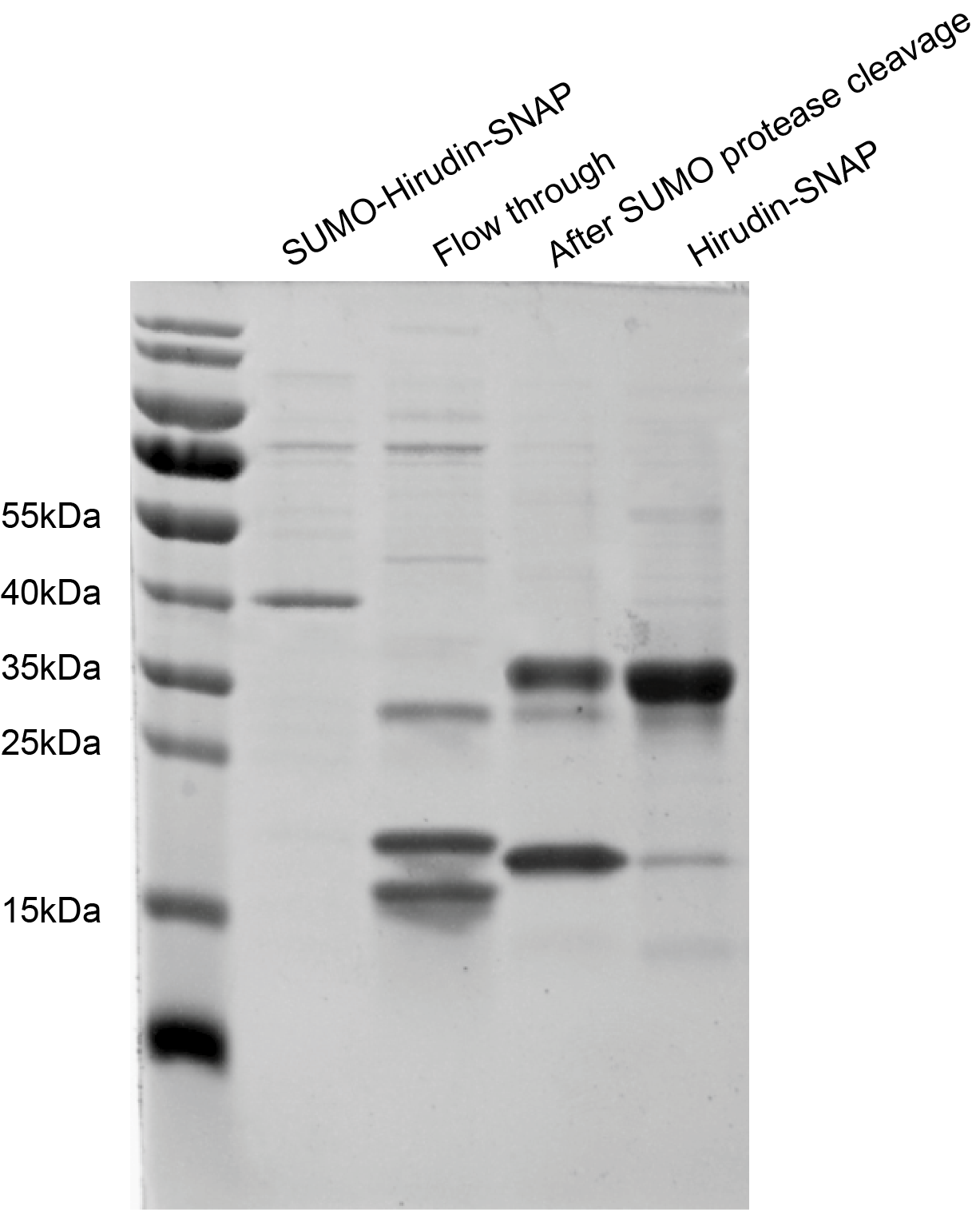

Supplement: Supplementary file 4 — Source Data [file 41467_2023_44607_MOESM4_ESM.zip › Uncropped blots.docx]
